# Biocompatible Cu/NiMo Composite Electrocatalyst for Hydrogen Evolution Reaction in Microbial Electrosynthesis; Unveiling the Self‐Detoxification Effect of Cu

**DOI:** 10.1002/advs.202309775

**Published:** 2024-03-29

**Authors:** Byeong Cheul Moon, Soyoung Kim, Young Yoon Jo, Jong Hyeok Park, Ja Kyong Ko, Dong Ki Lee

**Affiliations:** ^1^ Clean Energy Research Center Korea Institute of Science and Technology (KIST) Seoul 02792 Republic of Korea; ^2^ Center for Water Cycle Research Korea Institute of Science and Technology (KIST) Seoul 02792 Republic of Korea; ^3^ Department of Chemical and Biomolecular Engineering Yonsei‐KIST Convergence Research Institute Yonsei University Seoul 03722 Republic of Korea; ^4^ Division of Energy and Environment Technology KIST School University of Science and Technology Seoul 02792 Republic of Korea; ^5^ Graduate School of Energy and Environment Korea University Seoul 02841 Republic of Korea

**Keywords:** biocompatibility, CO_2_ utilization, H_2_‐evolving catalysts, microbial electrosynthesis, self‐detoxification

## Abstract

H_2_‐driven microbial electrosynthesis (MES) is an emerging bioelectrochemical technology that enables the production of complex compounds from CO_2_. Although the performance of microbial fermentation in the MES system is closely related to the H_2_ production rate, high‐performing metallic H_2_‐evolving catalysts (HEC) generate cytotoxic H_2_O_2_ and metal cations from undesirable side reactions, severely damaging microorganisms. Herein, a novel design for self‐detoxifying metallic HEC, resulting in biologically benign H_2_ production, is reported. Cu/NiMo composite HEC suppresses H_2_O_2_ evolution by altering the O_2_ reduction kinetics to a four‐electron pathway and subsequently decomposes the inevitably generated H_2_O_2_ in sequential catalytic and electrochemical pathways. Furthermore, in situ generated Cu‐rich layer at the surface prevents NiMo from corroding and releasing cytotoxic Ni cations. Consequently, the Cu/NiMo composite HEC in the MES system registers a 50% increase in the performance of lithoautotrophic bacterium *Cupriavidus necator* H16, for the conversion of CO_2_ to a biopolymer, poly(3‐hydroxybutyrate). This work successfully demonstrates the concept of self‐detoxification in designing biocompatible materials for bioelectrochemical applications as well as MES systems.

## Graphical abstract

Cu/NiMo composite H_2_‐evolving electrocatalysts greatly increase microbial electrosynthesis efficiency by enhancing H_2_ evolution performance and imparting superior biocompatibility via self‐detoxification which promotes selective 4‐electron O_2_ reduction and fast decomposition of H_2_O_2_, thereby enabling *Cupriavidus necator* H16 to efficiently produce poly(3‐hydroxybutyrate) from CO_2_ in pH‐neutral biological media.

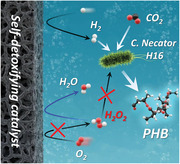

## 1 Introduction

The ever‐increasing concern for climate change and the need for carbon neutrality has resulted in an urgency to develop novel technologies for capturing and utilizing CO_2_.^[^ ^1^ ^]^ Among various CO_2_ conversion technologies, microbial electrosynthesis (MES), which combines electrochemical and biological approaches to convert CO_2_ into fuels and other useful chemicals through microbial fermentation,^[^ ^2^, ^3^ ^]^ has attracted considerable attention because microorganisms selectively produce complex carbon compounds from CO_2_ through the cellular metabolic process using electrons, transferred directly from electrodes or indirectly through electron mediators.^[^ ^4^, ^5^ ^]^ H_2_‐driven MES system utilizes H_2_ as an electron donor and H_2_‐oxidizing bacteria to convert CO_2_ to higher alcohols^[^ ^6^, ^7^ ^]^ and other polymeric substances,^[^ ^8^, ^9^ ^]^ employing expanded synthetic biology tools. H_2_‐oxidizing bacteria can be cultivated aerobically by utilizing in situ generated H_2_ during water electrolysis in the integrated water‐splitting electrochemical cell.^[^ ^10^, ^11^ ^]^ As bacteria accept electrons via H_2_ molecules, the performance of lithoautotrophic microbial fermentation in the MES system is closely related to the overall H_2_ production rate.^[^ ^12^, ^13^ ^]^

Carbonaceous materials have been widely used as an H_2_‐evolving catalyst (HEC) for the MES because of their proven compatibility with microorganisms.^[^ ^14^ ^]^ However, the slow kinetics of the H_2_ evolution reaction (HER) lowers the H_2_ evolution rate and limits microbial CO_2_ fixation efficiency. Therefore, the design and generation of novel high‐performance metallic HECs such as Ni,^[^ ^15^ ^]^ MoS_2_,^[^ ^16^ ^]^ CoP,^[^ ^6^ ^]^ and NiMoZn^[^ ^10^, ^17^ ^]^ has garnered considerable interest. Despite the excellent performance of metallic HECs, the biological incompatibility between microorganisms and metallic HECs leading to the generation of biologically harmful species during electrolysis is the major challenge toward achieving robust microbial fermentation performance in the MES system.^[^ ^14^, ^17^ ^]^ A key issue is the formation of H_2_O_2_, which is a representative reactive oxygen species generated during electrolysis and is detrimental to microbial metabolism.^[^ ^6^, ^10^ ^]^

**Figure 1:**
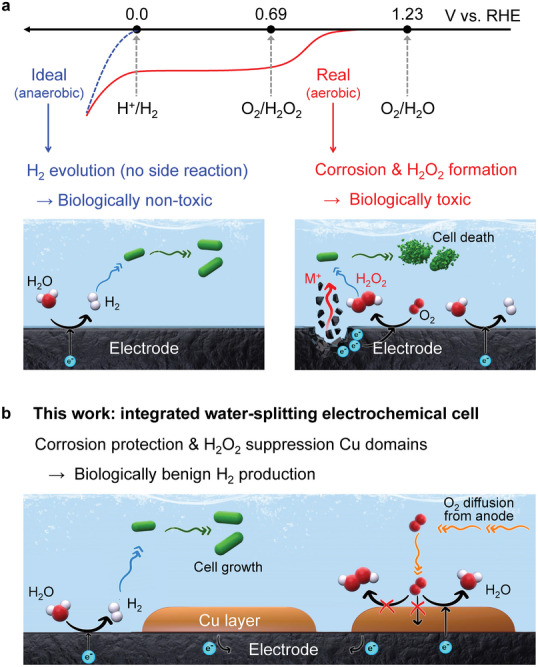
a) Electrochemical reactions occurring on the metallic H_2_ evolving catalyst (HEC) in an aqueous microbial fermentation environment under ideal (anaerobic) and real‐world (aerobic) conditions. b) Proposed working mechanism for biocompatible metallic HEC with Cu passivation layer.

**Figure** **1** schematically represents the electrochemical reactions occurring at the metallic HEC electrode under ideal and real conditions in an aqueous solution. In an ideal solution, H_2_ is selectively produced through the HER (2H^+^ + 2e^−^ → H_2_, *E* ^0^ = 0.00 V vs reversible hydrogen electrode potential, RHE) (Figure 1a). However, in a real‐world scenario, solutions contain dissolved O_2_, making the competing O_2_ reduction reactions (ORR) inevitable owing to their thermodynamically favorable reaction potentials. Particularly, H_2_O_2_ is generated either by 2‐electron ORR (O_2_ + 2H^+^ + 2e^−^ → H_2_O_2_, *E* ^0^ = 0.69 V vs RHE) or through corrosion of metals (M + 2H^+^ + O_2_ → M^2+^ + H_2_O_2_), which severely inhibits the cell growth. In addition, owing to their cytotoxic behavior, the elution of metal cations from transition metallic HECs also causes lethal stress on microorganisms.^[^ ^18^, ^19^ ^]^ To address these issues, there have been reports in literature such as embedding Ni nanoparticles in carbon nanotubes to prevent Ni leaching^[^ ^20^ ^]^ or wrapping microorganisms with metal‐organic frameworks to protect them from H_2_O_2_.^[^ ^21^ ^]^ Pre‐adaptation in the sub‐lethal concentration of H_2_O_2_ was also found to enhance the resistance of bacteria to oxidative stress with subsequent exposure to H_2_O_2_ to some extent.^[^ ^22^ ^]^ However, there was a limit to its application in environments where toxic species are continuously generated and accumulated during the reaction. Although Liu et al.^[^ ^6^ ^]^ developed a self‐healing mechanism to inhibit Co^2+^ dissolution during electrolysis using CoPi and CoP electrodes, studies on controlling in situ generated H_2_O_2_ are still lacking.

Herein, we report a concept of self‐detoxification toward biologically benign H_2_ production via the rational design of metallic HECs. We developed a biocompatible Cu/NiMo composite HEC to suppress and eliminate cytotoxic species, such as H_2_O_2_ and metal cations, during the MES reaction (Figure 1b). To investigate the proposed strategies, the intrinsic HER and ORR activities of metallic HECs were examined in a pH‐neutral electrolyte. The Cu/NiMo composite HEC rapidly decomposes the in situ generated H_2_O_2_ through sequential catalytic and electrochemical pathways, and the superficial Cu‐rich layer partially passivates the surface to prevent the underlying NiMo alloy from corroding and releasing Ni cations. Consequently, the Cu/NiMo composite HEC showed significantly improved HER activity and reduced H_2_O_2_ selectivity, resulting in a considerable enhancement in the performance of a bacterium, *Cupriavidus necator* H16 (*C. necator* H16), for converting CO_2_ to poly(3‐hydroxybutyrate) (PHB). Designing self‐detoxifying HECs using Cu modification represents a versatile approach to successfully employ metallic electrodes in bioelectrochemical applications by significantly improving their compatibility with microorganisms.

## 2 Results and Discussion

### 2.1 Characterization of Cu‐Modified HECs

**Figure 2:**
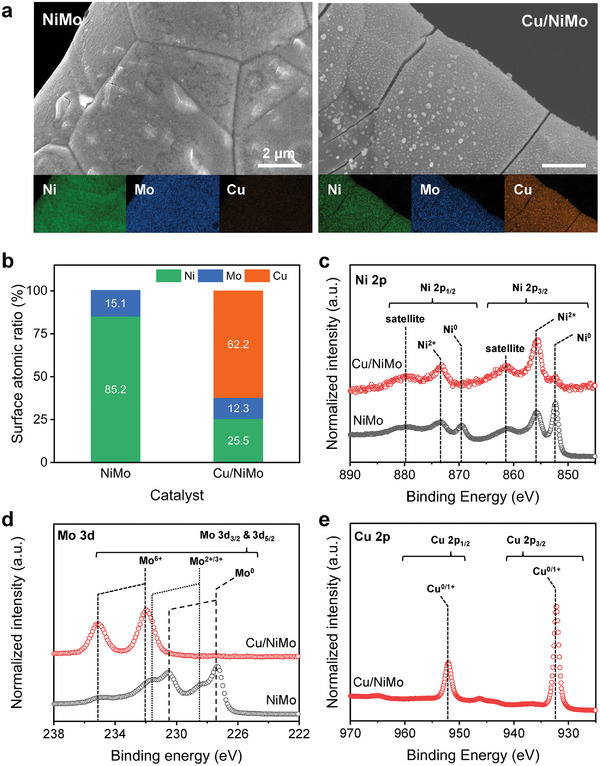
Structural characterization for NiMo and Cu/NiMo catalysts. a) FE‐SEM and EDS mapping images, b) atomic composition, and XPS spectra for c) Ni 2p, d) Mo 3d, e) Cu 2p.

To investigate the role of Cu modification on the metallic HEC, a transition metal alloy comprising Ni and Mo, namely NiMo and Cu‐modified NiMo (Cu/NiMo), was chosen as a model HEC. NiMo is an industrially relevant catalyst due to its excellent HER characteristics and has advantages compared to Co‐based catalysts because of relatively lower material cost and toxicity (Figure S19, Supporting Information). The electrodes were prepared by the electrochemical co‐deposition of metals on a Ni foam substrate (Figure S1, Supporting Information). The scanning electron microscopy (SEM) images show that the Ni foam substrates were fully covered with metallic films (**Figure** **2**a; Figure S2, Supporting Information). The grazing‐incidence X‐ray diffraction (XRD) patterns revealed that NiMo and Cu/NiMo have an amorphous nature, so the XRD patterns of as‐deposited and post‐annealed catalysts were compared to understand the structures (Figure S3, Supporting Information). The annealing temperature was determined by a thermal decomposition experiment using thermogravimetric analysis (TGA) under air (Figure S4, Supporting Information). As the temperature increased, NiMo showed two weight loss steps due to the evaporation of adsorbed water (range I) and the decomposition of surface metal hydroxides (range II) and weight gain while oxide formation (range III). Similarly, Cu/NiMo showed a weight loss by water evaporation (range I) and weight gain by Cu oxide formation (range III). These results support that NiMo has a low‐crystalline Ni_2_Mo structure, and Cu/NiMo is a composite material consisting of Ni_2_Mo and metallic Cu rather than a single Cu‐Ni‐Mo alloy. The elemental mapping image (Figure 2a) using energy dispersive spectroscopy (EDS) and the resulting surface atomic ratio (Figure 2b) showed that Cu partially covered the surface of Cu/NiMo. The X‐ray photoelectron spectroscopy (XPS) spectra for Ni 2p (Figure 2c) showed that the NiMo had both metallic Ni^0^ (852.6 eV) and Ni^2+^ (853.7 eV) states corresponding to 2p_1/2_ with a split distance of 17.3 eV, but the Cu/NiMo had dominant Ni^2+^ states. The Mo 3d spectra (Figure 2d) showed that NiMo consists of metallic Mo^0^ (227.3 eV), Mo^2+/3+^ (228.4 eV), and Mo^6+^ (232.4 eV) states corresponding to 3d_3/2_ with a split distance of 3.1 eV, whereas the Cu/NiMo has dominant Mo^6+^ states. This indicates that the co‐deposition of Cu induces partial oxidation of Ni and Mo to higher oxidation states, whereas metallic Cu^0^ (933 eV, split distance of 19.8 eV) is formed above the NiMo alloy (Figure 2e). Meanwhile, the weak satellite peak of Cu 2p (945 eV) and metal–oxygen bonds in the O 1s spectra (Figure S5, Supporting Information) implied that the metallic Cu covering the Cu/NiMo surface formed a thin Cu_2_O passivation film upon spontaneous oxidation in air. Raman spectra (Figure S6, Supporting Information) also provide an atomic arrangement and bonding nature of the NiMo and Cu/NiMo. Raman bands at 217 and 615 cm^−1[^ ^23^ ^]^ support the existence of a surface Cu_2_O layer, which is consistent with XPS results (Figure 2e). Raman scattering peaks at 284 cm^−1^ (Mo‐O_4_) and 334 cm^−1^ (O‐Mo‐O) indicate octahedral Mo‐O_6_ structures with Mo^6+^ states for Cu/NiMo, while NiMo has tetrahedral MoO_4_ structures with Mo^4+^ states (Figure 2d). The reason for the apparently different Mo oxidation states is a thermodynamic preference for the electroreduction of metal ions. During the electrodeposition of Cu/NiMo, metallic Cu and Ni_2_Mo phases are synthesized as composite materials. Cu^2+^, Ni^2+^, and MoO_4_^2^^−^ ions are simultaneously reduced by the cathodic potential, but the MoO_4_^2^^−^ is less likely to be reduced compared to Cu^2+^ (Cu^2+^/Cu, E^0^ = 0.34 V vs RHE) or Ni^2+^ (Ni^2+^/Ni, E^0^ = −0.26 V vs RHE) due to the low standard reduction potential (E^0^) of MoO_4_^2^^−^ (MoO_4_^2^^−^/Mo, E^0^ = −0.91 V vs RHE). In addition, mass transfer of MoO_4_^2^^−^ could be hindered either by Ni^2+^ or Cu^2+^ ions due to the lower E^0^ or large ionic radius resulting in the extended diffusion length. Electroreduction of MoO_4_^2^^−^ is kinetically impeded in the presence of Cu^2+^ for Cu/NiMo deposition, so that the Ni_2_Mo phase tends to be electron deficient for Cu/NiMo composite, and oxidation states of Mo for Cu/NiMo appears to be higher than those of NiMo. These findings are consistent with other reports on the NiCuMo medium‐entropy alloy catalyst with shorter Cu/Ni‐Mo bond formation.^[^ ^24^ ^]^

### 2.2 Electrochemical H_2_‐ and H_2_O_2_‐Evolving Properties

**Figure 3:**
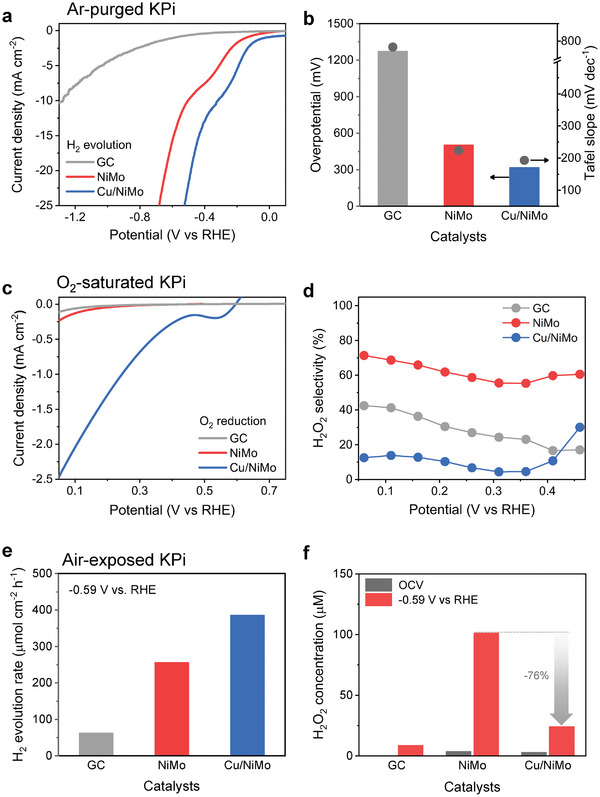
Electrochemical properties of GC, NiMo, and Cu/NiMo electrodes in a108 mm KPi at pH 7. a) Linear sweep voltammetry curves and b) comparison diagram of overpotential and Tafel slopes for H_2_ evolution reaction in Ar‐purged electrolyte. c) Rotating disk voltammetry curves and d) H_2_O_2_ selectivity for O_2_ reduction in O_2_‐saturated electrolyte. e) H_2_ evolution rates at −0.59 V versus RHE and f) H_2_O_2_ concentration after OCV for 10 min and chrono‐amperometry at −0.59 V versus RHE for 1 h in the integrated electrolysis cell within air‐exposed electrolyte.

The electrochemical HER activities were evaluated in Ar‐purged 108 mm potassium phosphate buffer (KPi) at pH 7, which is the optimal condition for the growth of the H_2_‐oxidizing bacterium *C. necator* H16. The composition of Cu/NiMo was optimized by varying the Cu concentration in the deposition solution (Figure S7, Supporting Information). The surface Cu coverage increased with higher Cu concentration, and the optimal Cu coverage was 64% (Figure S8, Supporting Information). Linear sweep voltammetry (LSV) curves in **Figure** **3**a showed that the HER activity of Cu/NiMo outperformed that of the NiMo and glassy carbon (GC). The electrochemical surface area (ECSA)‐corrected LSV curves were also plotted to examine the intrinsic HER activity by ruling out the extrinsic factors of surface area (Figures S9 and S10, Supporting Information). The Cu/NiMo still outperformed in the ECSA‐corrected LSV curve. The comparison diagram for the overpotential to achieve −10 mA cm^−2^ and the Tafel slope of electrodes in Figure 3b indicate that HER is thermodynamically and kinetically more favorable for the Cu/NiMo than the NiMo or GC. Electrochemical impedance spectroscopy (EIS) analysis indicated that the metallic Cu phase increases the electronic conductivity of the Cu/NiMo composite and decreases the charge‐transfer resistance of NiMo (Figure S11 and Table S1, Supporting Information).^[^ ^25^ ^]^ Following there is a trade‐off between HER properties and Cu surface coverage, it is plausible that NiMo acts as an active site and Cu acts as a promoter to improve electrical conductivity and optimize hydrogen bond energy.^[^ ^24^ ^]^

The electrochemical ORR activity was examined in an O_2_‐saturated 108 mm KPi at pH 7 using a rotating ring disk electrode (RRDE) technique. The electrodes were prepared by electrochemically co‐depositing HECs directly on the disk electrode of the RRDE. The Pt ring electrode was covered with chemically resistant Kapton tape to avoid metal contamination during the deposition. The LSV curves show that the Cu/NiMo has an outstanding ORR reactivity than the NiMo and GC (Figure 3c). Given that the reduction current is the sum of the 2‐electron ORR current for H_2_O_2_ evolution (O_2_ + 2H^+^ + 2e^−^ → H_2_O_2_, *E* ^0^ = 0.69 V vs RHE) and the 4‐electron ORR current for water evolution (O_2_ + 4H^+^ + 4e^−^ → 2H_2_O, *E* ^0^ = 1.23 V vs RHE), the H_2_O_2_ selectivity by the undesirable 2‐electron ORR was determined using the ring current and collection efficiency of 0.35 (Figure 3d). The average H_2_O_2_ selectivity of the Cu/NiMo (11%) was much lower than that of the NiMo (62%) and GC (29%) in the potential region between 0.06 and 0.46 V versus RHE, indicating that the high ORR currents of Cu/NiMo mostly originated from the water production current via 4‐electron ORR (Figure S12, Supporting Information).

The HER performance was examined with the HECs deposited on a Ni foam substrate and a gas‐tight cell connected to the gas chromatograph (Figure 3e). Before the HER, the electrolyte (108 mm KPi at pH 7) was exposed to air without Ar or O_2_ purging. While the NiMo produced H_2_ at a rate of 256 µmol cm^−2^ h^−1^, the Cu/NiMo showed a 1.5‐fold enhancement in the H_2_ evolution rate with a value of 386 µmol cm^−2^ h^−1^. The amount of H_2_O_2_ produced during electrolysis was also quantified using the iodometric titration method (Figure S13, Supporting Information) under unbiased (at open‐circuit voltage, OCV) and biased conditions (at −0.59 V vs RHE), respectively (Figure 3f). When the electrode was immersed in the electrolyte for 10 min at OCV, a small amount of H_2_O_2_ (<4 µm) was chemically generated by the corrosion reaction in which the metallic HEC reacts with dissolved O_2_. When a constant potential of −0.59 V versus RHE was applied for 1 h, the H_2_O_2_ concentration steeply increased to 101 µm for the NiMo. By contrast, only 24 µm of H_2_O_2_ was recorded for the Cu/NiMo, which was 76% lower than that for the NiMo. This clearly demonstrated that the Cu/NiMo effectively suppressed H_2_O_2_ evolution in an integrated cell with an O_2_‐dissolved electrolyte.

**Figure 4:**
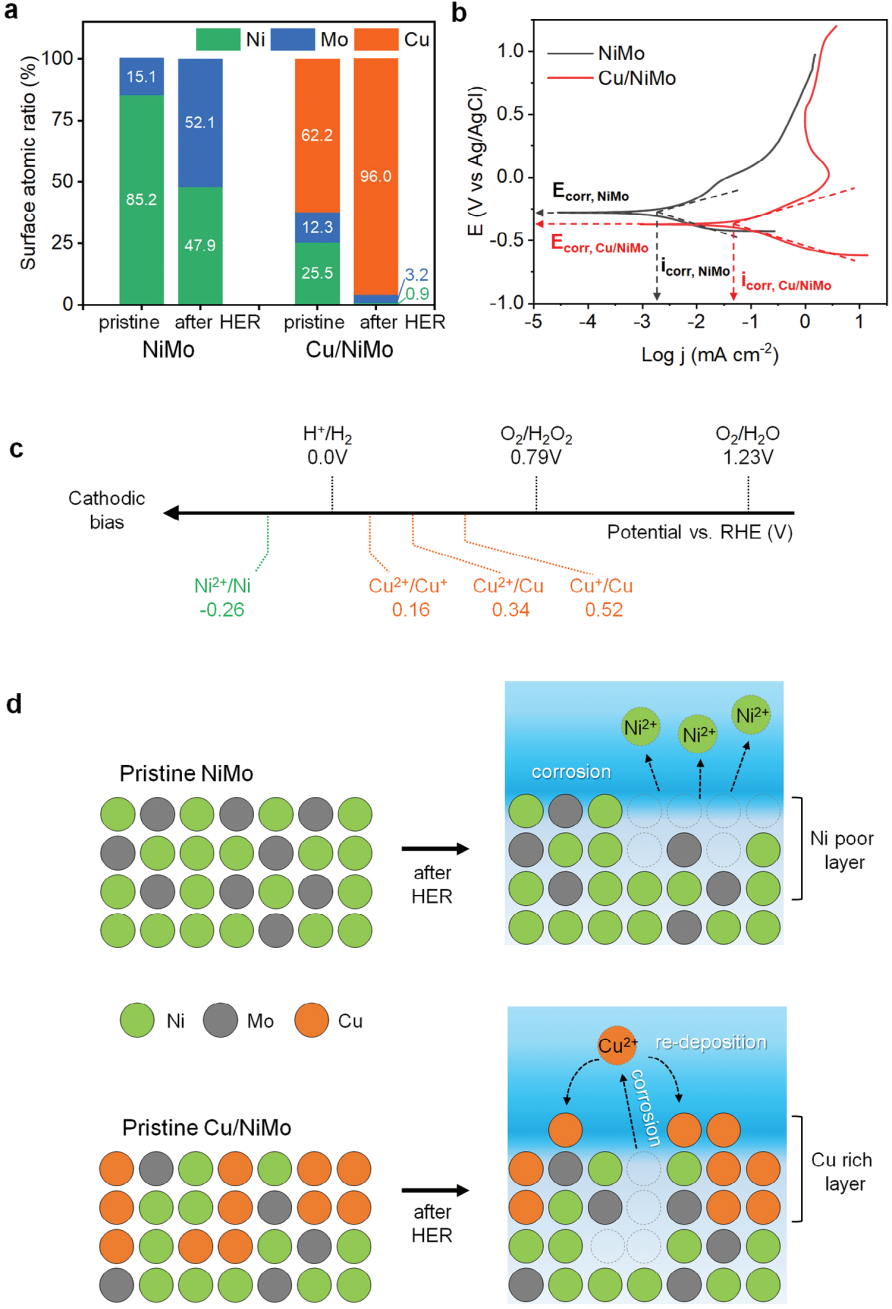
The Cu effect on the surface reconstruction of Cu/NiMo during the HER. a) Atomic composition of NiMo and Cu/NiMo surface before and after HER at −20 mA cm^−2^ in a 108 mm KPi at pH 7 for 200 h. b) Anodic scan of potentiodynamic polarization curves for NiMo and Cu/NiMo in the KPi, where *E*_corr_ is corrosion potential and *i*_corr_ is corrosion current. c) Standard reduction potentials for Ni and Cu cations with H^+^ and O_2_. d) Schematic illustration of corrosion and surface reconstruction mechanisms of NiMo and Cu/NiMo after HER in a pH‐neutral electrolyte.

The stability of electrodes was evaluated with the HECs deposited on a Ni foam substrate in an integrated cell, where the cathode and anode are not divided by the membrane. The repetitive cyclic voltammetry (CV) scan over 1000 cycles was conducted at potentials ranging from 0.05 to −0.55 V versus RHE. The Cu/NiMo retained HER activity with a marginal potential shift of 12 mV at −10 mA cm^−2^ (Figure S14a, Supporting Information). The chronopotentiometry curves for the HER at −20 mA cm^−2^ with the NiMo and Cu/NiMo electrodes showed a negligible degradation rate of approximately 0.1 mV h^−1^ over 200 h (Figure S14b, Supporting Information). The SEM images of electrodes after the HER showed that large, bumpy grains were formed at the surface of Cu/NiMo, while minor changes with a fine crack appeared on the surface of NiMo (Figure S15, Supporting Information). The compositional change by the HER was also dramatic on the Cu/NiMo; the Cu became dominant, accounting for 96%, and the Ni content was drastically reduced from 26% to less than 1% (**Figure** **4**a). In addition, the compositional changes of Cu/NiMo composite HEC in the bulk were investigated by cross‐sectional SEM analyses. The SEM and EDS mapping images (Figure S16a, Supporting Information) show that the thickness of the Cu/NiMo catalyst is 900 nm and each element (Cu, Ni, and Mo) is well distributed over the bulk catalyst layer. The line scan result (Figure S16b, Supporting Information) supports that surface Cu concentration near the surface is higher than that of bulk. However, no significant increase in the Cu concentration near the surface was observed in the XPS (Figure 4a), which indicates that the formation of a Cu‐rich layer is limited to the shallow surface (<30 nm).

These indicate that the surface of Cu/NiMo is reconstructed into a metallic Cu‐rich surface during the HER. To understand the morphological and compositional changes, the corrosion properties of electrodes were examined using a potentiodynamic polarization experiment (Figure 4b). By the Cu modification, the corrosion potential (*E*_corr_) of NiMo was shifted to the cathodic direction by 94 mV and the corrosion current (*i*_corr_) was increased by 46‐fold, which indicates that the Cu/NiMo is more susceptible to corrosion (Table S2, Supporting Information). However, the dissolved Cu^2+^ cations are easily re‐deposited onto the surface under the cathodic biased condition because of the higher standard reduction potential of Cu^2+^ (Cu^2+^/Cu, E^0^ = 0.34 V vs RHE) compared to that of Ni^2+^ (Ni^2+^/Ni, E^0^ = −0.26 V vs RHE) and HER (H^+^/H_2_, *E* ^0^ = 0.00 V vs RHE) as shown in Figure 4c. This enables dynamic reconstruction of the Cu‐rich surface layer in the Cu/NiMo during the HER, whereas metallic Ni at the NiMo surface is oxidized into Ni^2+^ cations, and the electrons are then transferred to the surface to reduce dissolved O_2_ in the electrolyte into H_2_O_2_, which makes the Ni‐poor layer (Figure 4d). Meanwhile, the change in the chemical state of the HECs after the HER was negligible (Figure S17, Supporting Information).

### 2.3 Microbial Production of PHB in the Bioelectrochemical Reactor

**Figure 5:**
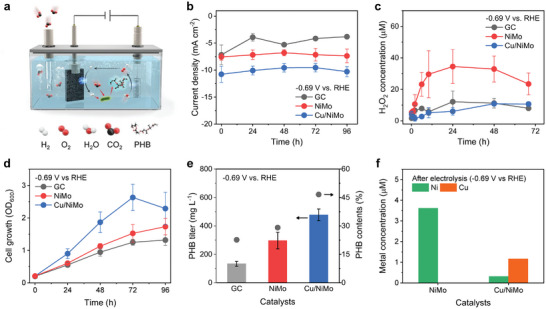
Microbial fermentation performance at −0.69 V versus RHE in the minimal medium. a) Schematic illustration of MES system, b) current densities, c) H_2_O_2_ concentration during the initial 70 h, d) microbial cell growth curves with OD_600_ for 96 h, e) PHB titer and contents, and f) metal concentration after electrolysis. Each experiment was performed in triplicate, and the error bars indicate the standard deviation of the mean of triplicate values.

The Cu/NiMo possessing enhanced HER with suppressed 2‐electron ORR was then assessed for the microbial production of PHB in an integrated bioelectrochemical reactor (**Figure** **5**a). MES experiments were conducted for 96 h in the presence of *C. necator* H16 in the minimal medium, which contains minimum nutrients such as trace elements and nitrogen sources required for microbial cell growth (see Experimental Section for details). Throughout the MES reaction, CO_2_ was continuously supplied to the minimal medium at 10 mL min^−1^. When a potential of −0.69 V versus RHE was applied to the electrodes, the Cu/NiMo showed a higher H_2_‐evolving current density compared to the GC and NiMo (Figure 5b). Since the reaction environment for MES is different from that of pure KPi used for electrochemical characterization of HECs, the amount of H_2_O_2_ produced in the minimal medium was evaluated over reaction time. The amount of H_2_O_2_ was monitored and quantified using a fluorometric assay kit after separating the microorganisms from the solution to avoid inaccurate titration. After 24 h of reaction, the NiMo produced 34 µm of H_2_O_2_ (Figure 5c), which exceeds a level that significantly reduces cell viability as previously reported by Torella et al.^[^ ^10^ ^]^ The H_2_O_2_ concentration decreased slowly as the reaction proceeds, but still maintained a high concentration over 20 µm. On the other hand, relatively low amounts of H_2_O_2_ were generated for the GC (6.8 µm) and Cu/NiMo (2.4 µm) for 24 h and did not exceed 12 µm during the overall reactions.

The cell mass in the minimal medium was also evaluated by measuring the optical density at 600 nm (OD_600_), as depicted in Figure 5d. The cell growth rate on Cu/NiMo was found to be significantly faster than that on the NiMo and GC electrodes. It reached a maximum at 72 h with an average OD_600_ of 2.6, which was nearly two times higher than that of the NiMo. Stationary phases were observed at 72 h where the rate for cell growth and death became equal, and the OD_600_ decreased after 72 h. During the initial 24 h, the cell growth rate for the NiMo was similar to that of the GC while the NiMo is a superior HER catalyst with 50% higher HER current. This is because an excess amount of H_2_O_2_ generated from NiMo inhibits cell growth. However, the final OD_600_ of the GC after 96 h was not much greater than unity due to the poor H_2_ evolving properties of the GC even with the few amount of H_2_O_2_ accumulation. These indicate that the critical factors determining cell growth are H_2_‐evolving as well as H_2_O_2_‐suppressing properties of the HECs. The target product, PHB, produced by *C. necator* H16, was quantified using high‐performance liquid chromatography (Figure 5e), and the PHB titer of Cu/NiMo (487 mg L^−1^) was found to be 1.5 times higher than that of the NiMo (300 mg L^−1^). The performance of the MES system was further investigated at different potentials to determine the optimal operating conditions for the best‐performing Cu/NiMo (Figure S18a–c, Supporting Information). When the applied potential increased from −0.39 to −0.69 V versus RHE, the cell mass measured by OD_600_ gradually increased with higher current densities, indicating that active H_2_ evolution promoted cell growth. However, abrupt cell death was observed at the highest potential of −0.79 V versus RHE after 48 h, which could be attributed to violent H_2_ evolution accompanied by the generation of heat and bubbles. The cell growth for the GC electrode also showed that the cell mass increased with the current density for H_2_ evolution at higher potentials, but the PHB productivity was limited by the inferior HER properties of the GC electrode (Figure S18d–f, Supporting Information). To better understand the microbial fermentation performance in the MES, further work should be conducted on the microenvironmental changes such as nutrient concentration or local pH.

Despite the efficient H_2_ evolution and H_2_O_2_ suppression ability of the Cu/NiMo, the dissolution of transition metal cations into the electrolyte via corrosion can lead to cytotoxicity and hamper the growth of microorganisms.^[^ ^19^, ^26^ ^]^ To evaluate the effect of dissolved metal ions from the HECs on the cell growth, the metal concentration in the electrolytes after the electrolysis at −0.69 V versus RHE for 3 h was quantified using inductively coupled plasma‐optical emission spectrometry (Figure 5f). The toxicity of transition metals composing the HECs was examined using a spot assay (Figure S19, Supporting Information). Ni^2+^ was observed to be toxic at concentrations above 20 µm, consistent with a previous report.^[^ ^6^ ^]^ However, Cu^2+^ was found to be considerably safe and hardly cytotoxic to microorganisms in the concentration range of 0–100 µm. The amount of dissolved Ni and Cu cations from the HECs was well below the high toxicity levels. Moreover, the dissolution of Ni cation from the Cu/NiMo was noticeably reduced owing to the dominant Cu phase covering the surface. Since Cu covers the surface more than 60% (Figure 2b), the exposed surface area of NiMo is smaller than NiMo, so the amount of Ni dissolution should be lower. In addition, the Cu phase becomes a local anode while increasing bulk conductivity and transferring electrons to the HER active NiMo phase (local cathode), so the corrosion of NiMo is hindered by cathodic protection by sacrificial Cu anode. However, the high standard reduction potential of Cu enables surface reconstruction with a Cu‐rich layer with facile Cu re‐deposition (Figure 4d; Figure S15, Supporting Information). This finding clearly demonstrated remarkably improved biocompatibility of the Cu/NiMo composite HEC by preventing the dissolution of Ni cation during the electrolysis.

### 2.4 Mechanism Study on Biologically Benign H_2_ Production via Self‐Detoxification

**Figure 6:**
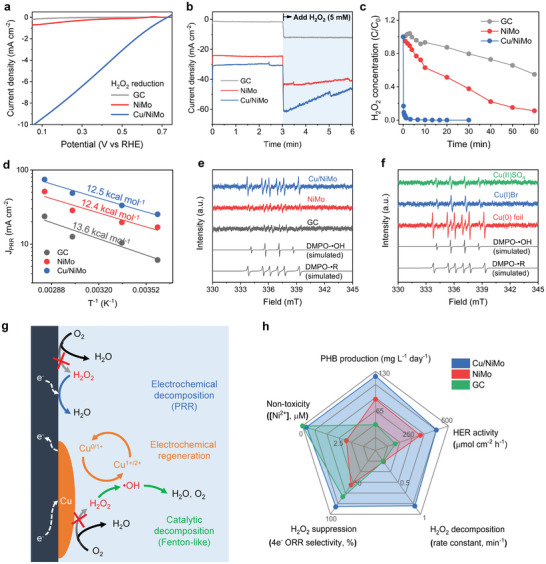
Cu redox‐mediated self‐detoxification mechanism of Cu/NiMo. a) Rotating disk voltammetry curves for H_2_O_2_ reduction, b) chronoamperometry at −0.59 V versus RHE with the addition of 5 mm H_2_O_2_ after 3 min, c) time‐course H_2_O_2_ decomposition curves at −0.59 V versus RHE, and d) Arrhenius plot on the PRR current with 5 mm H_2_O_2_ at −0.59 V versus RHE at various temperatures of 278, 298, 331, and 353 K and the activation energy for H_2_O_2_ reduction. All electrolysis was performed in a 108 mm KPi at pH 7. EPR spectra of radical intermediates trapped by DMPO in a 5 mm H_2_O_2_ dissolved Ar‐purged KPi with (e) all electrodes and (f) various Cu species. The simulated radicals for DMPO–**^•^** R (g = 2.00561; A_N_ = 15.3 G; A_H_ = 23 G) and DMPO‐**^•^** OH (g = 2.00561; A_N_ = 15 G; A_H_ = 15 G) adducts are also shown. g) Proposed mechanism of the self‐detoxification process on the Cu/NiMo electrode, f) radar plot determining the efficiency of the MES system with five parameters, namely PHB production, HER activity (H_2_ production rate), low metal toxicity (Ni^2+^ concentration after electrolysis for 3 h), H_2_O_2_ suppression (4‐electron ORR selectivity), and H_2_O_2_ decomposition (H_2_O_2_ decomposition rate constant, k) for the electrodes.

Even trace amounts of H_2_O_2_ are highly lethal to microorganisms; therefore, accumulated H_2_O_2_ in the reaction solution needs to be minimized. The use of Pt as an anode would help to decrease H_2_O_2_ content in the electrolyte because the Pt anode aids in the decomposition of H_2_O_2_ into O_2_ via 2‐electron oxidation reaction (H_2_O_2_ → O_2_ + 2H^+^ + 2e^−^, *E* ^0^ = 0.69 V vs RHE, Figure S20, Supporting Information).^[^ ^27^ ^]^ Nevertheless, controlling the in situ generated H_2_O_2_ near the cathode is still the most important factor in preserving the viability of microorganisms because the large amount of O_2_, which is generated by the water oxidation reaction at the anode, is continuously diffused throughout the electrolysis cell, resulting in a continuous generation of H_2_O_2_ at the cathode surface via 2‐electron ORR. In the cathode reaction, H_2_O_2_ can be reductively decomposed into water on metallic surfaces such as Pt, Ni, Co, and Cu^[^ ^28^, ^29^ ^]^ through the electrochemical peroxide reduction reaction (PRR) via 2‐electron reduction (H_2_O_2_ + 2H^+^ + 2e^−^ → 2H_2_O, *E* ^0^ = 1.79 V vs RHE). The PRR activity of the HECs was examined using the rotating disk electrode (RDE) technique (**Figure** **6**a). The rotating disk voltammetry curves of the HECs were recorded in Ar‐purged 108 mm KPi at pH 7 to which 5 mm H_2_O_2_ was added. The Cu/NiMo showed a high PRR current, approximately 6–10 times higher than that of the NiMo and GC. The PRR current response of the HECs deposited on a Ni foam substrate was also examined under similar conditions. When 5 mm H_2_O_2_ was injected into the electrolyte, a sudden increase in the cathodic current was observed owing to the PRR currents for H_2_O_2_ decomposition and a local pH drop by H_2_O_2_ (Figure 6b). The increment in the current for Cu/NiMo (31 mA cm^−2^) was much higher than those for the NiMo (14 mA cm^−2^) and GC (10 mA cm^−2^), which was proportional to the PRR activities of the HECs. The amount of H_2_O_2_ generated during the decomposition reaction at −0.59 V versus RHE was also monitored through UV–vis absorption spectroscopy in a time‐course manner (Figure 6c). The decomposition curves show that the time required to reach half the initial H_2_O_2_ concentration for Cu/NiMo was much shorter than that for the NiMo and GC. Surprisingly, the kinetic constant for H_2_O_2_ decomposition for the Cu/NiMo (0.845 min^−1^) was 60 times higher than that for the NiMo (0.014 min^−1^) (Figure S21, Supporting Information). In the Arrhenius plots (Figure 6d), while PRR currents of the Cu/NiMo are 1.6 times higher than those of the NiMo, the activation energy required for PRR of Cu/NiMo (12.4 kcal mol^−1^) and NiMo (12.5 kcal mol^−1^) was almost identical each other. These results imply the existence of another pathway for H_2_O_2_ decomposition on the Cu/NiMo rather than an electrochemical PRR.

To investigate the plausible H_2_O_2_ decomposition mechanism other than the electrochemical pathway at the Cu/NiMo, the radical intermediates were analyzed and characterized via electron paramagnetic resonance (EPR). The radicals generated during the decomposition reaction were detected using 5,5‐dimethyl‐1‐pyrroline N‐oxide (DMPO) as the spin‐trapping molecule. When the Cu/NiMo electrode was exposed to Ar‐purged 108 mm KPi with 5 mm H_2_O_2_ at the OCV, ten lines of signals were promptly generated and assigned to the mixed signals of DMPO–hydroxide (^•^OH)^[^ ^30^, ^31^ ^]^ and carbon‐centered radical (DMPO–^•^R)^[^ ^32^ ^]^ adducts. The EPR intensity of the Cu/NiMo was remarkably higher than those of the NiMo and GC (Figure 6e), indicating that H_2_O_2_ decomposition proceeded vigorously on Cu through the Fenton‐like pathways^[^ ^33^ ^]^ to produce ^•^OH radicals. Because the Fenton‐like reaction could be caused by various Cu species, a control experiment was conducted by the addition of Cu foil (Cu^0^), CuBr (Cu^+^), and CuSO_4_ (Cu^2+^) in the electrolyte containing 5 mm H_2_O_2_ (Figure 6f). The quartet EPR peaks corresponding to DMPO‐^•^OH arose in every condition, indicating that the H_2_O_2_ decomposition could be caused by both metallic and ionic Cu. It is well known that mono‐ and divalent Cu decompose H_2_O_2_ into OH^−^ or O_2_ through homogeneous reactions (Equations (1), (2), (3), (4)),^[^ ^34^, ^35^ ^]^ and zero‐valent Cu directly decomposes adsorbed H_2_O_2_ into H_2_O or O_2_ on the heterogeneous Cu through catalytic reactions (Equations (5), (6), (7)).^[^ ^36^, ^37^, ^38^ ^]^

Homogeneous pathway:

(1)

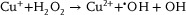

(2) $$Cu^{2+}+H_{2}O_{2}\to Cu^{+}{+}^{•}\mathrm{OOH}+H^{+}$$

(3) $$Cu^{+}{+}^{•}\mathrm{OH}\to Cu^{2+}+OH^{-}$$

(4) $$Cu^{2+}{+}^{•}\mathrm{OOH}\to Cu^{+}+H^{+}+O_{2}$$

Heterogeneous pathway:

(5)

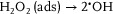



(6)

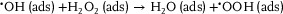

(7) $$2^{•}\mathrm{OOH}\left(\mathrm{ads}\right)\to H_{2}O_{2}\left(\mathrm{ads}\right)+O_{2}$$

We note that the sextet EPR peaks corresponding to the DMPO–^•^R adduct gradually decreased and disappeared within 30 min after H_2_O_2_ was completely decomposed in the Cu/NiMo at −0.59 V versus RHE (Figure S22, Supporting Information). Although ^•^OH radicals were detected for all the Cu species, carbon‐centered radicals were generated only from the zero‐valent Cu as a reaction product between adsorbed ^•^OH radicals and carbon contaminants.^[^ ^39^ ^]^ Therefore, the presence of DMPO–^•^R adduct provides indirect evidence that Cu decomposes H_2_O_2_ through a heterogeneous Fenton reaction as well as a homogeneous reaction by dissolved Cu cations.

Figure 6g schematically depicts the proposed mechanism of the self‐detoxifying HEC electrode. Cu/NiMo composite HEC forms a superficial Cu‐rich layer with corrosion and subsequent re‐deposition of Cu, which passivates the underlying NiMo alloy from reacting with O_2_ to evolve H_2_O_2_ and prevents the release of toxic Ni cations. In addition, the Cu‐rich layer efficiently suppresses the electrochemical generation of H_2_O_2_ by altering the ORR kinetics toward the 4‐electron pathway to selectively reduce O_2_ to water and subsequently remove the undesirable H_2_O_2_ via two different mechanisms. First, H_2_O_2_ is electrochemically decomposed via 2‐electron PRR because the superficial Cu‐rich layer destabilizes the O─O bond of adsorbed H_2_O_2_^[^ ^40^ ^]^ and reduces to water by transferring electrons to the adsorbed OH.^[^ ^41^, ^42^ ^]^ Second, metallic Cu catalytically decomposes H_2_O_2_ via heterogeneous Fenton‐like reactions, and Cu cations eluted from the electrode contribute to homogeneous Fenton‐like reactions. The homogeneous reaction could involve the corrosion of Cu in the reaction media; however, the released Cu cations are easily re‐deposited on the surface under cathodic bias to rebuild the Cu‐rich layer. This electrochemical regeneration of the Cu^[^ ^43^, ^44^ ^]^ layer was supported by EDS and XPS results obtained after long‐term electrolysis (Figures S15 and S17, Supporting Information). Meanwhile, the Cu/NiMo retained superior HER activity even after the formation of Cu‐rich layers, which is presumed to be the increased surface area of the Cu/NiMo by the dissolution of Cu cation despite the decreased surface coverage by HER‐active NiMo. As the Cu cations are virtually non‐toxic (Figure S19, Supporting Information) and easily re‐deposited under the cathodic bias (Figure S15, Supporting Information), Cu greatly contributes to the H_2_O_2_ decomposition without harming the cell viability. Five critical parameters were derived to elucidate the relationship between the electrochemical properties of the electrodes and MES system efficiency in the radar plot (Figure 6h). The Cu/NiMo electrode outperformed the NiMo and GC in terms of HER activity (i.e., H_2_ evolution rate), H_2_O_2_ suppression (selectivity toward the 4‐electron ORR), and H_2_O_2_ decomposition (rate constant, k) while maintaining the low toxicity of metal cations (Ni^2+^ dissolution) in the electrolyte. To the best of our knowledge, our design on the self‐detoxifying HEC using Cu/NiMo composite resulted in one of the highest PHB production rates of 0.120 g L^−1^ day^−1^ in the H_2_‐driven MES system, compared to previously reported electrodes (Table S3, Supporting Information).

## 3 Conclusion

This study demonstrates a simple, but versatile protocol for rationally designing Cu‐modified metallic HECs for enhanced microbial electrosynthesis of PHB from CO_2_ using *C. necator* H16. A self‐detoxifying metallic Cu/NiMo composite HEC was successfully developed by Cu modification, which significantly improved the biocompatibility of metallic Cu/NiMo HEC by suppressing and eliminating the cytotoxic species generated in the MES reaction media. The superficial Cu‐rich layer protected the underlying NiMo from corroding and releasing toxic cations. Mechanistic studies further established that Cu contributes to the decomposition of reactive oxygen species in a comprehensive manner, through catalytic and electrochemical pathways. Using the self‐detoxifying HEC in the MES reaction resulted in biologically benign H_2_ production, while significantly enhancing the production of target chemicals. The study provides a novel and facile strategy for the design of biocompatible electrodes, and our approach can be potentially applied to the production of electrodes in tailor‐made bioelectrochemical applications.

## 4 Experimental Section

### Materials

Nickel chloride hexahydrate (NiCl_2_∙6H_2_O), sodium molybdate dihydrate (Na_2_MoO_4_∙2H_2_O), zinc chloride (ZnCl_2_), copper chloride dihydrate (CuCl_2_∙2H_2_O), sodium bicarbonate (NaHCO_3_), sodium pyrophosphate (Na_4_P_2_O_7_), formic acid (CH_2_O_2_), potassium iodide (KI), sodium hydroxide (NaOH), ammonium molybdate tetrahydrate ((NH_4_)_6_Mo_7_O_24_ ∙4H_2_O), and potassium hydrogen phthalate (C_8_H_5_KO_4_, KHP) were purchased from Sigma–Aldrich, and 95% H_2_SO_4_ was purchased from Junsei Chemical (Japan). *C. necator* H16 was purchased from the Korean Collection for Type Cultures (KCTC) and routinely cultivated at 30 °C and 200 rpm. To grow *C. necator* H16 strains electroautotrophically, the cells grown in rich Luria–Bertani (LB) broth for 24 h were transferred into a fresh minimal medium with 10 g L^−1^ fructose in a 250 mL flask and incubated for 24 h. For pre‐adaptation of *C. necator* H16 to autotrophic growth, cells were then cultured in serum bottles filled with a total 150 kPa of mixture gas (O_2_:H_2_:CO_2_ = 20:70:10, Airkorea corporation, South Korea) and further incubated under autotrophic conditions. The cultured cells were centrifuged at 4200 rpm for 10 min and washed twice with a minimal medium. The harvested cells were then suspended in 100 mL of the minimal medium in a bioelectrochemical reactor at an optical density of 600 nm (OD_600_) of 0.2. The minimal medium consists of 6.74 g L^−1^ Na_2_HPO_4_·7H_2_O, 1.5 g L^−1^ KH_2_PO_4_, 0.2 g L^−1^ (NH_4_)_2_SO_4_, 80 mg L^−1^ MgSO_4_·7H_2_O, 1 mg L^−1^ CaSO_4_·2H_2_O, 0.56 mg L^−1^ NiSO_4_·7H_2_O, 0.4 mg L^−1^ FeC_6_H_5_O_7_, and 200 mg L^−1^ NaHCO_3_.

### Preparation of Electrodes

The HECs were prepared using modified electrochemical co‐deposition methods^[^ ^45^, ^46^ ^]^ (Figure S1, Supporting Information). Briefly, Ni foam substrates (30 × 10 mm; 1 mm thickness) were pre‐cleaned in a 2 m HCl solution in an ultrasonication bath for 15 min to eliminate innate surface Ni oxides, washed with distilled water and ethanol, and dried under an N_2_ stream. Then, the HECs were deposited on Ni foam with a constant current of −100 mA cm^−2^ for 15 min in a two‐electrode configuration using a Pt mesh as the counter electrode. The electrodeposition solution was prepared by dissolving 40 mm NiCl_2_∙6H_2_O, 20 mm Na_2_MoO_4_∙2H_2_O, and 0.1 mm ZnCl_2_ in 0.89 m NaHCO_3_ and 0.13 m Na_4_P_2_O_7_. To prepare Cu‐modified NiMo (Cu/NiMo), *x* mM (*x* = 5, 10, 20, and 40) of CuCl_2_∙2H_2_O was additionally dissolved in the solution. To promote the co‐deposition of multiple metals, 18.5 mm formic acid was added to the electrolyte before deposition. The prepared electrodes were then activated in 10 m KOH for 15 h to selectively dissolve the Zn metals from HECs to increase the surface area.

### Electrochemical Measurement

All electrochemical measurements were conducted using a potentiostat (VSP‐3e, Biologic, France) in a three‐electrode configuration with a Pt mesh counter electrode and an Ag/AgCl (filled with 3 m KCl) reference electrode. All linear sweep voltammetry (LSV) measurements were performed at a scan rate of 10 mV s^−1^ in an undivided cell unless otherwise stated. The HER properties were evaluated in 108 mm potassium phosphate (KPi) electrolyte at pH 7, and the electrolyte was purged with Ar (99.999%) for more than 30 min before each experiment to isolate the reaction current from the oxygen reduction reaction (ORR) from dissolved oxygen in the electrolyte. LSV curves for the HER were subjected to 85% *iR*‐correction, where *R* is the solution resistance obtained via electrochemical impedance spectroscopy (EIS). The electrochemical surface area (ECSA) of HECs was also estimated by dividing the double‐layer capacitance (*C*_dl_) of electrodes into specific capacitance (*C*_s_) in an alkaline solution. The *C*_dl_ of electrodes was obtained by the slope of current to scan rates plots in the potential range of non‐faradaic current in 0.1 m KOH.

The ORR properties were evaluated using a rotating ring disk electrode (RRDE) setup (RRDE‐3A model 2325, ALS, Japan) in a four‐electrode configuration with a glassy carbon (GC) disk electrode of the RRDE as the working electrode, a Pt ring electrode as the second working electrode, a Pt mesh as the counter electrode, and an Ag/AgCl reference electrode. The RRDE was carefully polished before use, and the HECs were electrodeposited on a GC disk as described above. Deposition time was controlled to less than 3 min to prevent the thick catalyst film from peeling off from the disk while rotating. LSV scans were performed in an O_2_‐saturated 108 mm KPi electrolyte at a rotating speed of the RRDE of 1600 rpm. The ring potential was set to 1.2 V versus RHE to detect H_2_O_2_ generated on the disk electrode. The electrolyte was sparged with O_2_ (99.999%) at least 30 min before measurement. H_2_O_2_ selectivity and average electron transfer number (*n*) were calculated using the following equations:

(8) $$Selectivity\;\;\left(\%\right)=\;200\times \frac{\left(\frac{I_{\mathrm{ring}}}{N}\right)}{\left(I_{\mathrm{disk}}+\frac{I_{\mathrm{ring}}}{N}\right)};\;n\;=\;4\times \frac{\left|I_{\mathrm{disk}}\right|}{\left(I_{\mathrm{disk}}+\frac{I_{\mathrm{ring}}}{N}\right)}$$

where *I*_ring_ and *I*_disk_ are the ring and disk currents of the RRDE electrode, respectively, and *N* is the collection efficiency (0.35). The peroxide reduction reaction (PRR) was conducted using a rotating disk electrode (RDE) set up in a three‐electrode configuration with a GC disk electrode of the RDE as the working electrode, Pt wire as the counter electrode, and Ag/AgCl as the reference electrode. The RDE was polished before use, and the electrode was prepared by the same method as that used for preparing the RRDE. LSV scans were performed in Ar‐saturated 108 mm KPi with 5 mm H_2_O_2_ at the rotating speed of the RDE of 1600 rpm. Electrochemical H_2_O_2_ decomposition was conducted using chronoamperometry at a constant potential of −0.59 V versus RHE in Ar‐saturated 108 mm KPi with 5 mm H_2_O_2_, and HEC electrodes deposited on Ni foam were used to reveal the exact current responses in the MES system. The H_2_O_2_ decomposition kinetics were analyzed at different temperatures of 278, 298, 331, and 353 K, and the activation energy for peroxide reduction was calculated using the Arrhenius equation:

(9) $$log\left(i_{\mathrm{PRR}}\right)\;=\frac{-E_{a}}{RT}\;+A_{0}$$

where *i*_PRR_ is the PRR current, *A*_0_ is the prefactor, *E*_a_ is the activation energy for the PRR, *R* is the gas constant, and *T* is the temperature. *i*_PRR_ was obtained by subtracting the HER current from the total current at −0.59 V versus RHE.

### Bioelectrochemical Measurement

A custom‐made single‐chamber bioelectrochemical reactor was used in this study. A sterile 150 mL glass reactor filled with 100 mL of minimal medium was sparged with 5% CO_2_ gas (balanced with 95% N_2_) at 10 mL min^−1^ using a glass sparger. The bioelectrochemical reactions were conducted in a temperature‐controlled incubator at 30 °C using a potentiostat (WMPG1000Le8, WonAtech, Korea) in a three‐electrode configuration with the prepared NiMo and Cu/NiMo electrodes as the working electrodes, coiled Pt wires as a counter electrode and Ag/AgCl as a reference electrode. As a control group, a flat GC plate was used for a working electrode due to its biological compatibility and excellent durability. The geometrical area of electrodes was controlled to be 3 cm^2^. The NiMo and Cu/NiMo electrodes were pre‐conditioned by cyclic voltammetry (CV) in the potential region of 0.6 to −0.9 V versus RHE before the reaction to reduce the air‐oxidized surfaces.

### Characterization

Morphological and elemental analyses were performed using field‐emission scanning electron microscopy (FE‐SEM, Hitachi, Regulus 8230, Japan) at an accelerating voltage of 15 kV. Cross‐sectional images of the Cu/NiMo composite film were obtained by focused ion beam‐assisted SEM (FIB‐SEM, FEI Helios G4 HX, USA), and the Pt layer was coated above the films to protect the film during ion beam milling. The thin‐film X‐ray diffraction patterns were obtained using a grazing incidence X‐ray diffractometer (D/max2500/PC, Rigaku, Japan) with a Cu‐Kα radiation source at 18 kW (60 kV, 300 mA). For the XRD measurement, the HECs were deposited on a Ti foil substrate. The chemical states were investigated by X‐ray photoelectron spectrometry (XPS, K‐alpha, Thermo Scientific, USA) with an Al‐Kα micro‐focused monochromator at a beam current of 30 mA. Thermal stability was analyzed by a thermogravimetric analyzer (TGA, TA Discovery TGA 550, USA) from 25 to 900 °C at a ramping rate of 10 °C min under air atmosphere. Raman scattering spectra were obtained by Raman spectrometer (Renishaw in Via Raman Microscope, U.K.). Electron paramagnetic resonance (EPR) spectra were obtained using an EPR spectrometer (Jeol JES‐FA 200 X‐band, 9.45 GHz, Japan). The measurement parameters were as follows: microwave power, 5 mW; magnetic field modulation, 0.01 mT; modulation frequency, 100 kHz; and sweep width, 10 mT. All experiments were conducted at room temperature, and radical intermediates were analyzed using a Jeol LC‐11 flat glass capillary sample tube (capillary volume: 100 µL) with 5,5‐dimethyl‐1‐pyrroline N‐oxide (DMPO) as a spin trap molecule. Spot assays were performed to investigate the cytotoxicity of the Ni and Cu cations. Pre‐cultures of *C. necator* H16 were washed with the minimal medium and suspended to an initial OD_600_ of 0.2. The suspension was diluted tenfold serially to 10^−4^ and spotted on a minimal medium plate containing each toxicant. The spotted plates were then incubated in a pressure vessel containing a gas mixture of 20% of O_2_, 10% of CO_2_, and N_2_ balance for five days. The H_2_O_2_ concentration in the bioelectrochemical reactor was quantified using a fluorometric assay kit (Sigma–Aldrich). Fluorescence intensity was measured using a Spark multimode reader (Tecan Group Ltd., Switzerland) equipped with a fluorescence excitation/emission filter pair (540 ± 20 nm/590 ± 8 nm) using 96‐well black plates with a clear and flat bottom.

The amount of H_2_O_2_ was quantified using a standard curve ranging from 0 to 10 µm. In addition, the H_2_O_2_ concentration during the H_2_O_2_ decomposition reaction was quantified using an iodometric titration method.^[^ ^47^ ^]^ Briefly, aqueous solution A containing KI (66 g L^−1^), NaOH (2 g L^−1^), and (NH_4_)_6_Mo_7_O_24_∙4H_2_O (0.2 g L^−1^) and aqueous solution B containing KHP (20 g L^−1^) were prepared. Solutions A and B (1 mL each) were mixed with 1 mL of H_2_O_2_ and kept in the dark for 30 min. The absorption spectra of the mixed solution were obtained using a custom‐modified flow injection analysis‐UV–vis (FIA‐UV–vis) measurement system (Agilent, USA). Absorbance was measured at 350 nm, and the H_2_O_2_ concentration was calculated using a linear titration curve. PHB was quantified using crotonic acid according to the method of Law and Slepecky with modification.^[^ ^48^ ^]^ The cells were harvested by centrifugation for 10 min at 4200 rpm and lyophilized using a freeze dryer (Operon, Korea). After the dried cells were transferred to a 1.5 mL microcentrifuge tube, 0.5 mL of 95% H_2_SO_4_ was added to the cell pellet, and the mixture was incubated at 368 K for 1 h. The solutions were analyzed by high‐performance liquid chromatography (Agilent Technology 1260 Infinity, USA) with a HIPLEX‐H column (300 × 7.7 mm, Agilent Technology, USA) using a UV–vis detector and 5 mm H_2_SO_4_ as the mobile phase at 0.6 mL min^−1^. The dry cell weight (DCW) was calculated based on a pre‐determined standard curve (1 OD_600_ = 0.448 g DCW L^−1^).^[^ ^6^, ^49^ ^]^

## Conflict of Interest

The authors declare no conflict of interest.

## Supporting information

Supporting Information

## Data Availability

The data that support the findings of this study are available from the corresponding author upon reasonable request.
