# Supplementary material for: Biocompatible Cu/NiMo Composite Electrocatalyst for Hydrogen Evolution Reaction in Microbial Electrosynthesis; Unveiling the Self‐Detoxification Effect of Cu
Source: Adv Sci (Weinh). 2024 Mar 29;11(22):2309775. doi: 10.1002/advs.202309775 (PMC11165482; doi:10.1002/advs.202309775)
Supplement: Supplementary file 1 — Supporting Information [file ADVS-11-2309775-s001.pdf]

ADVANCED SCIENCE
Open Access

Supporting Information

for *Adv. Sci.*, DOI 10.1002/advs.202309775

Biocompatible Cu/NiMo Composite Electrocatalyst for Hydrogen Evolution Reaction in Microbial Electrosynthesis; Unveiling the Self-Detoxification Effect of Cu

Byeong Cheul Moon, Soyoung Kim, Young Yoon Jo, Jong Hyeok Park, Ja Kyong Ko and Dong Ki Lee**

Supporting Information

Biocompatible Cu/NiMo Composite Electrocatalyst for Hydrogen Evolution Reaction in Microbial Electrosynthesis; Unveiling the Self-Detoxification Effect of Cu

Byeong Cheul Moon, Soyoung Kim, Young Yoon Jo, Jong Hyeok Park, Ja Kyong Ko, and Dong Ki Lee**

B. C. Moon, S. Kim, Y. Y. Jo, J. K. Ko, D. K. Lee
Clean Energy Research Center, Korea Institute of Science and Technology (KIST), Seoul 02792, Republic of Korea
E-mail: dnklee@kist.re.kr, jkko@kist.re.kr

B. C. Moon
Center for Water Cycle Research, Korea Institute of Science and Technology (KIST), Seoul 02792, Republic of Korea

J. H. Park, D. K. Lee
Department of Chemical and Biomolecular Engineering, Yonsei-KIST Convergence Research Institute, Yonsei University, Seoul, 03722, Republic of Korea

J. K. Ko
Division of Energy and Environment Technology, KIST School, University of Science and Technology, Seoul 02792, Republic of Korea

D. K. Lee
Graduate School of Energy and Environment, Korea University, Seoul 02841, Republic of Korea

Figure S1. Schematic illustration on the fabrication procedure for electrodes using three-dimensional (3D) Ni foam substrate.

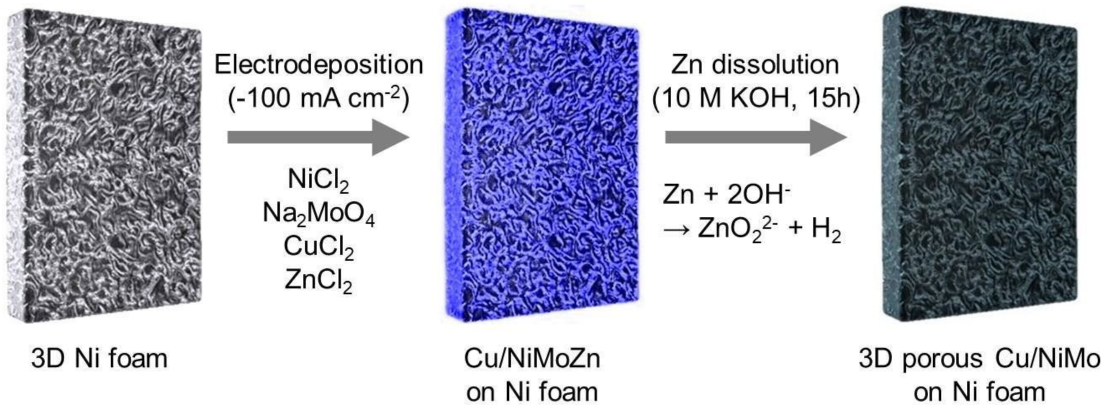

Notes: After electrochemical co-deposition of hydrogen evolving catalyst (HEC) on Ni foam, the electrodes were pre-activated in 10M KOH to leach out the Zn and increase surface area of electrodeposited electrodes (NiMo and Cu/NiMo). When electrodes were immersed in concentrated KOH solution, Zn reacts with hydroxide (OH^-) anions and leaches out in the form of ZnO_2^{2-} anion by generating H_2 gas.

Figure S2. Morphological characterization of electrodes. FE-SEM images of (a,b) pristine Ni foam, (c) NiMo, and (d) Cu/NiMo electrodes.

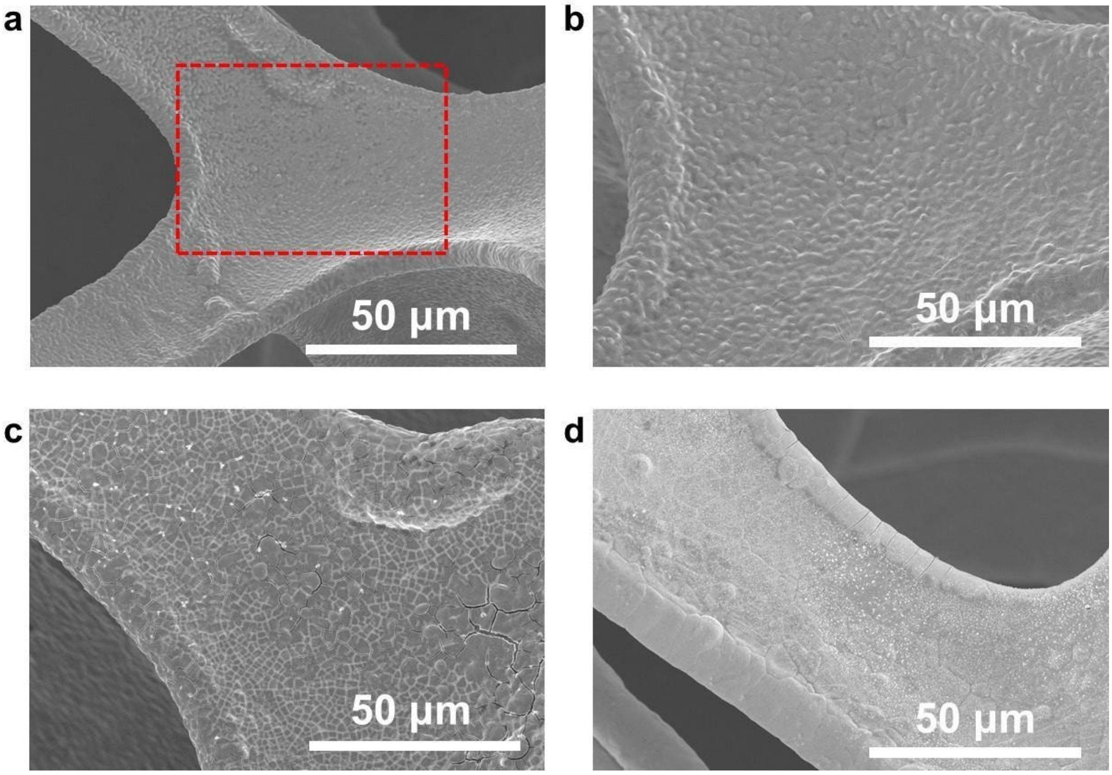

Figure S3. Grazing-incidence XRD patterns for (a) NiMo and (b) Cu/NiMo catalyst film deposited on Ti foil.

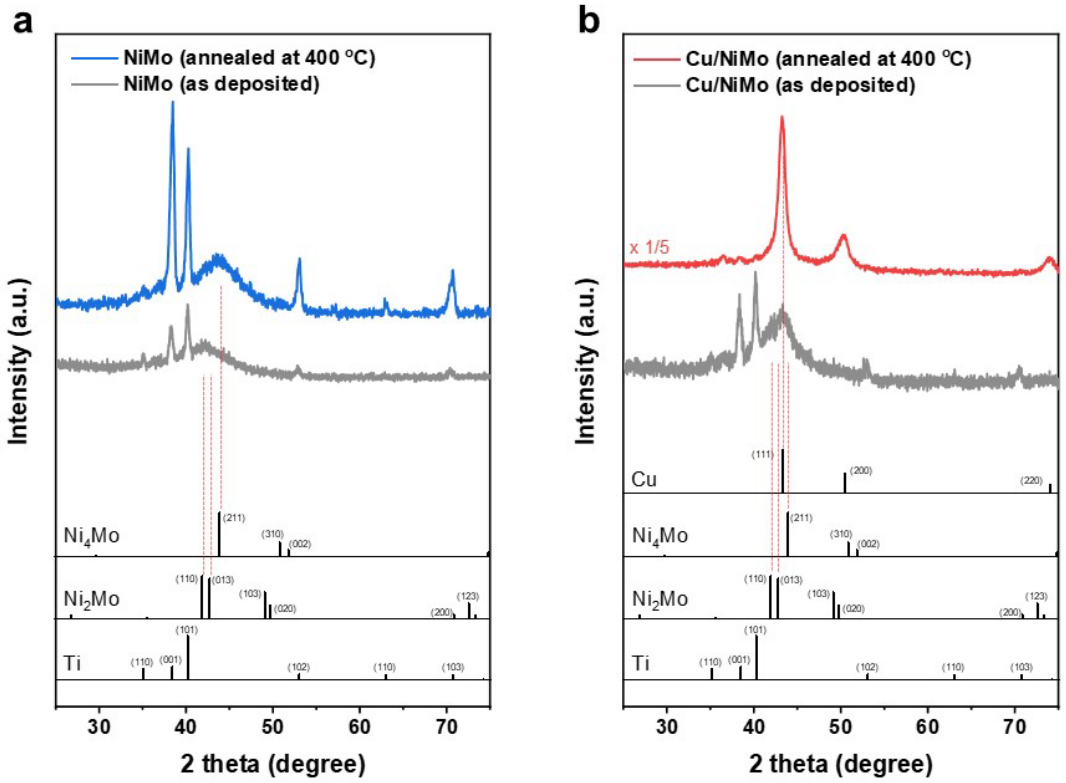

Note : Since as-deposited NiMo and Cu/NiMo have amorphous structure, the catalysts were annealed at 400 °C under inert Ar atmosphere to increase the crystaillinity without changing orignial structures as determined by the thermal degradation experiment (**Fig. S4**). Post-annealed NiMo showed slightly improved crystallinity and a shift in the main peak (43.9°) indicating stable Ni_4Mo structure in consistent with the crystalline NiMo alloy[1]. Post-annealed Cu/NiMo showed strong peak (43.3°) indicating (111) face of metallic Cu (PDF #04-0836). The XRD patterns for Ni_2Mo (mp-784630) are simulated patterns from the structural information from the Materialsproject.[2]

Figure S4. Thermogravimetric analysis (TGA) curve of (a,b) NiMo and (c,d) Cu/NiMo and Cu under air.

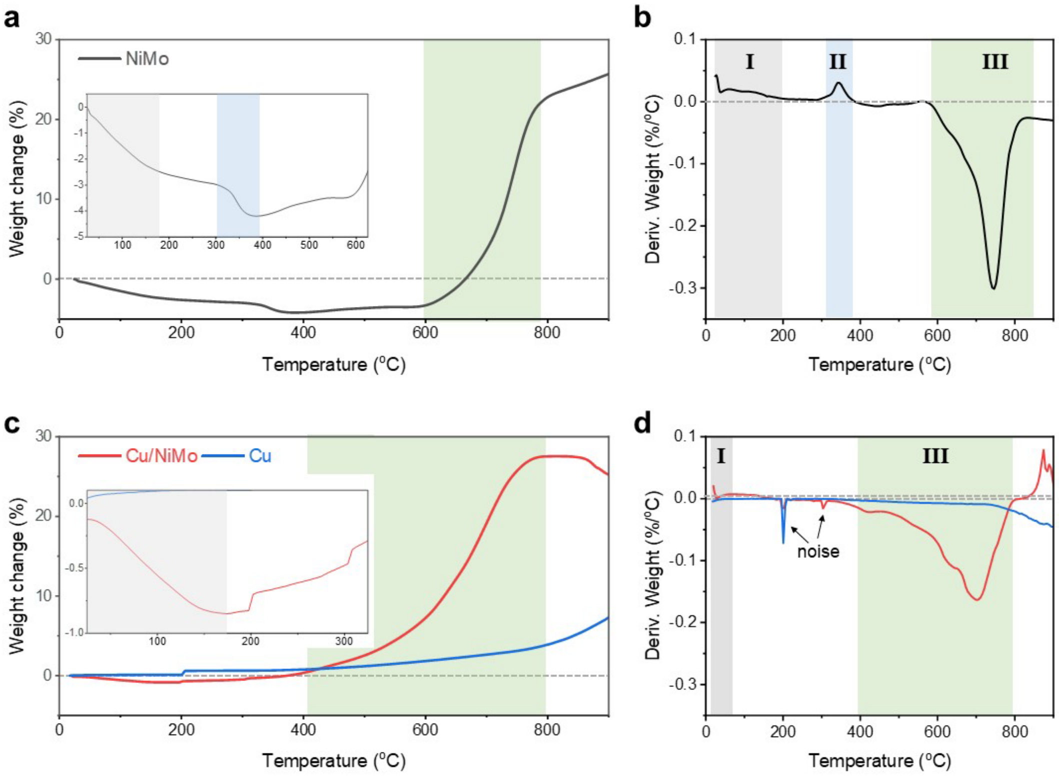

Note : Range I indicates weight loss due to the evaporation of adsorbed water molecules on the surface. Range II is only observed for NiMo, which is originated from the decomposition of metal hydroxides structures and formation of crystalline Ni_4Mo structures (**Fig S3**). Due to the oxide lyaer formation, the range III appears above 600 °C for NiMo , while the range III starts at 400 °C for Cu/NiMo due to copper oxides formation.

Figure S5. XPS spectra of (a) O 1s and (b) survey scan for the NiMo and Cu/NiMo.

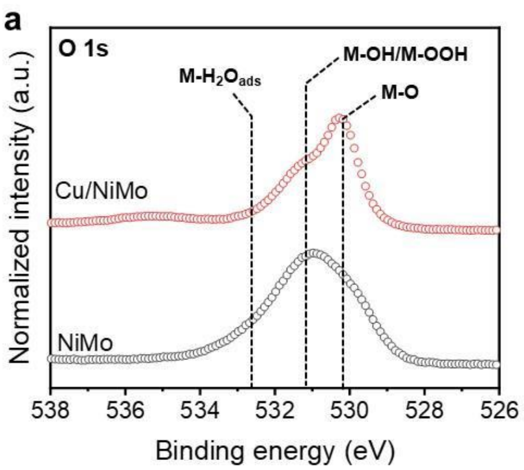

Note : (A) O 1s spectra show that the electrodes are covered with oxides or hydroxides when exposed to air. (B) Survey scan of electrodes clearly shows the elements that consist of each electrodes.

Figure S6. Raman spectra of NiMo and Cu/NiMo.

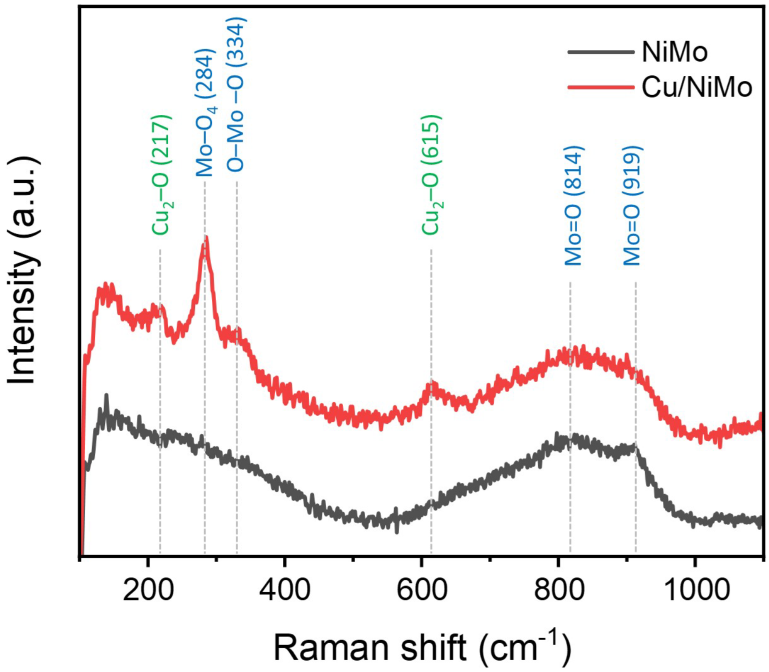

Figure S7. (a) Cyclic voltammetry curves and (b) comparison diagram of Cu/NiMo with different Cu concentrations in the deposition solution. (c) Linear scan voltammetry curves for Cu/NiMo (Cu_{40}/NiMo) with diffrent deposition times.

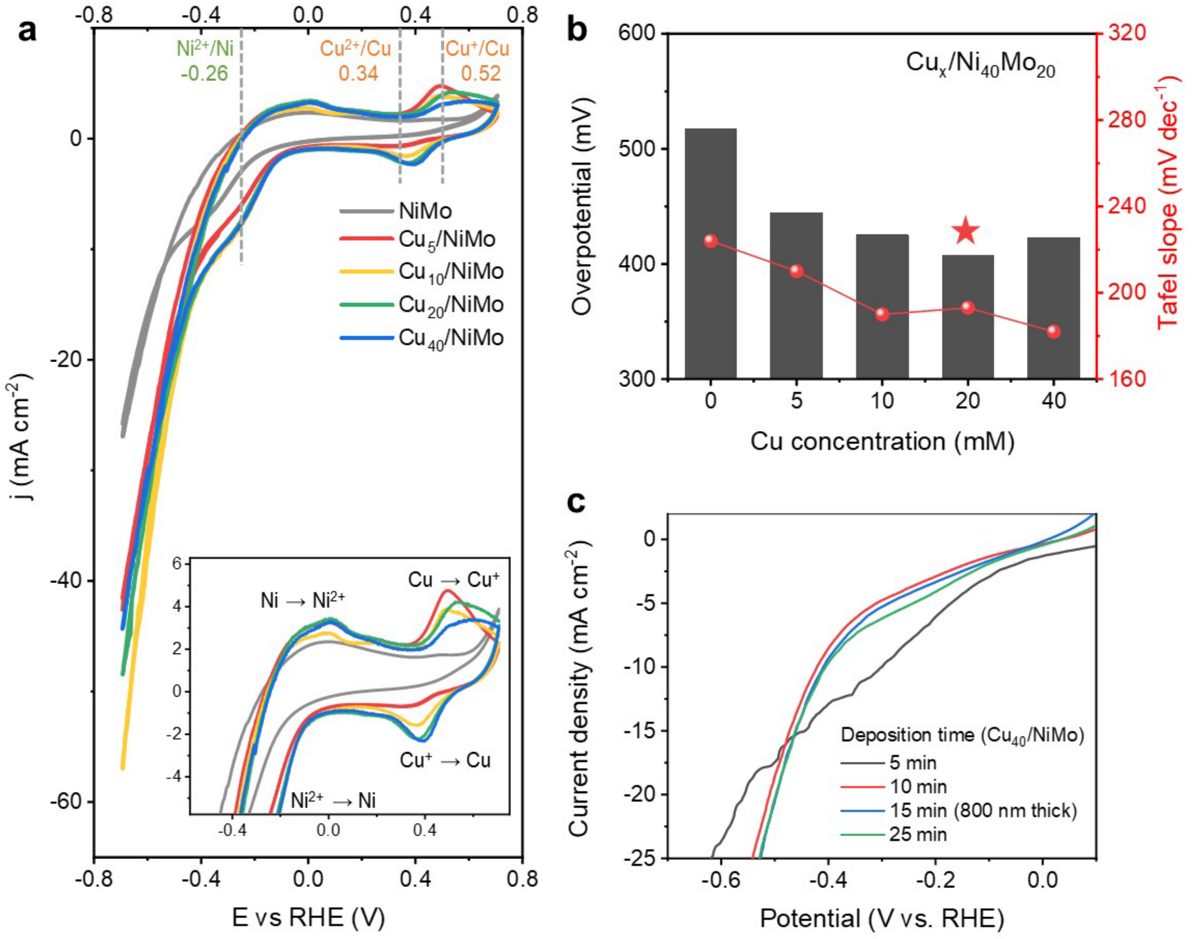

Note: The HER activity of Cu/NiMo electrodes were compared, and the overpotential and Tafel slope were changed with different Cu concentration in the deposition solution. The overpotential to reach -10 mA cm^{-2} decreased and Tafel slope for the HER was lowest at 20 mM of Cu, thereby the optimal composition for Cu/NiMo were prepared at compositional ratio of metals in deposition solution with Ni:Mo:Zn:Cu by 40:20:0.1:20 mM. In the cyclic voltammetry curves, redox peaks from redox couple of Ni^{2+}/Ni and Cu^{+}/Cu were shown and the peak intensity of Cu^{+}/Cu couple tends to increase with higher Cu concentration. In the cathodic scan, Cu^{+} reduction (0.37 V vs. RHE) and Ni^{2+} reduction (-0.31 V vs. RHE) peaks are observed. In the anodic scan, Ni oxidation (-0.01 V vs. RHE) and Cu oxidation (0.49 V vs. RHE) peaks are observed.

Figure S8. Morphological characterization of Cu/NiMo electrodes with different Cu concentration in the deposition solution. (a) FE-SEM images and (b) surface Cu coverage estimated by EDS.

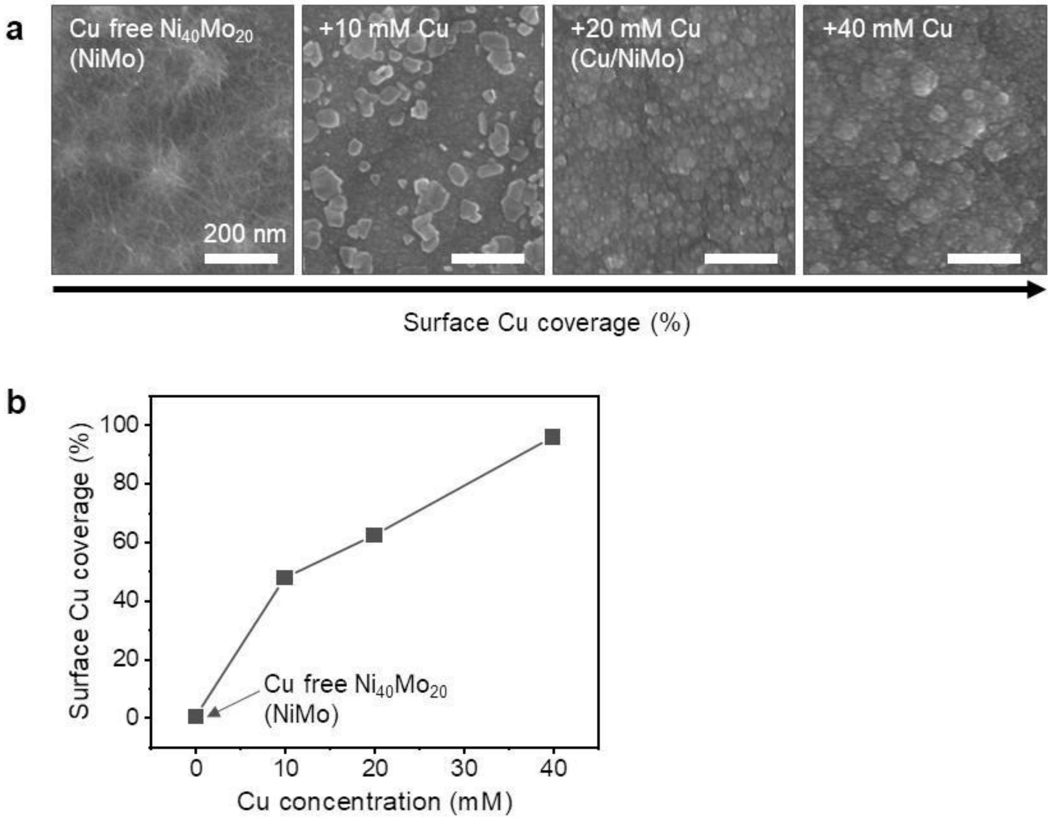

Figure S9. ECSA analysis of HEC electrodes. CV scans of (a) GC, (b) NiMo and (c) Cu/NiMo electrodes, and (d) ΔJ vs scan rate plots with different scan rates from 20 to 200 mV s^{-1} in a 0.1M KOH. (e) Table for electrochemical double-layer capacitance (C_{dl}), specific capacitance (C_s), and ECSA of HEC electrodes.

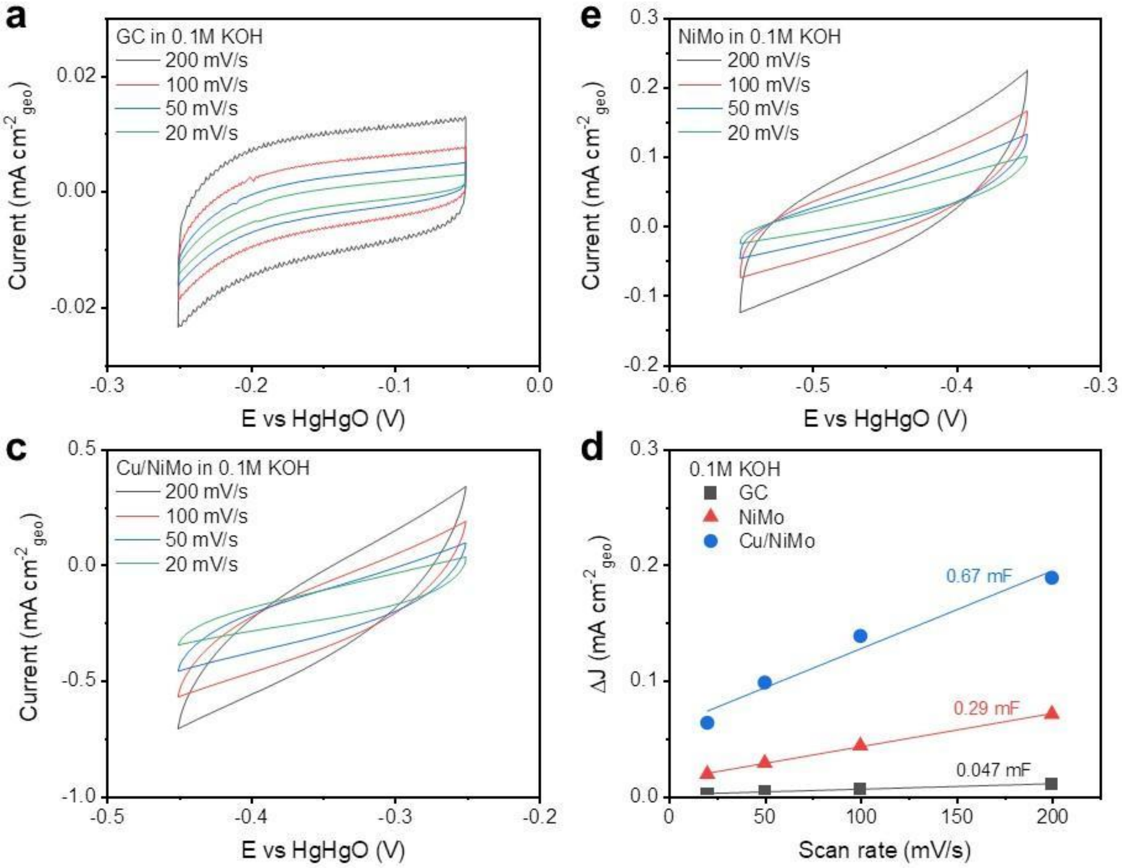

e

HEC electrodes	C_{dl} (μF)	C_s (μF cm^{-2}_{geo})	ECSA (cm^2)
**GC**	47	22	2.1
**NiMo**	290	30	9.6
**Cu/NiMo**	670	~90	7.4

Note: ECSA was estimated by dividing the C_{dl} into the C_s, and the C_s of NiMo and Cu/NiMo was calculated by linear combination of C_s [3] for each element based on the surface atomic ratio determined by XPS (**Fig 2b**).

Figure S10. ECSA-correctred LSV curves for the HECs in a pH-neutral 108 mM KPi.

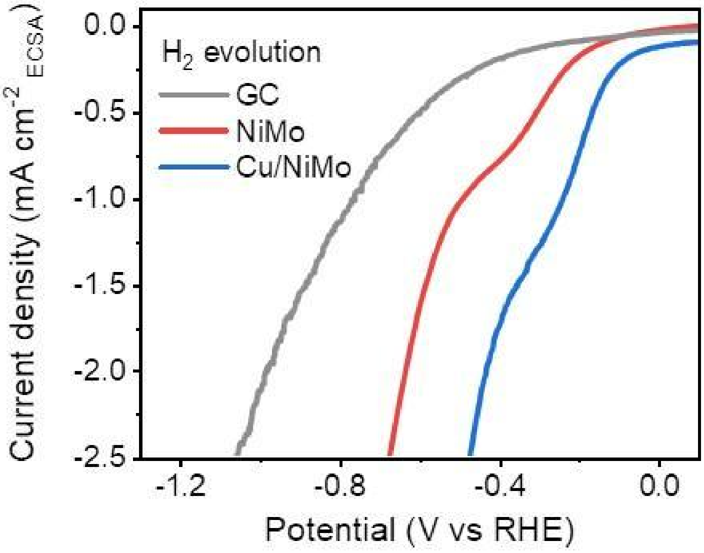

Figure S11. Nyquist plot of electrochemical impedance spectroscopy (EIS) at open circuit voltage in the 0.1 M KPi. EIS was measured in the frequency range of 100 kHz to 10 mHz, and fitted with a 3RC equivalent circuit where R is resistance (R) and C is constant phase element (CPE).

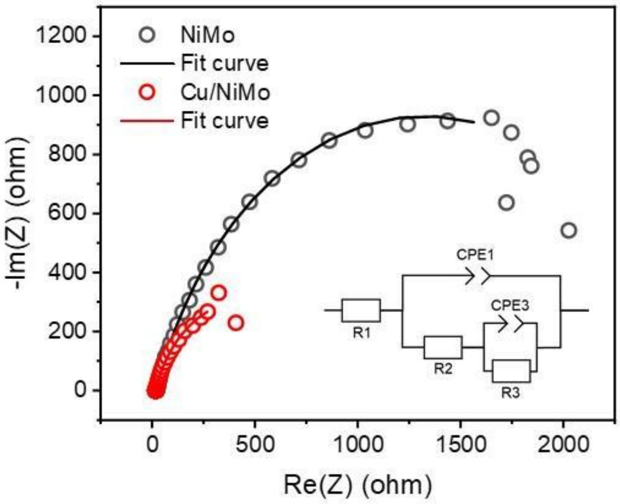

Table S1. EIS parameters fitted with an equivalent circuit.

Electrodes	R1 (ohm)	R2 (ohm)	R3 (ohm)	CPE1 (F)	CPE3 (F)	χ^2
NiMo	20.8	2583	30.7	6.1E-4	2.2E-6	0.164
Cu/NiMo	16.3	703	104	0.012	3.1E-8	0.074

Figure S12. Average electron transfer numbers for ORR in O_2-saturated 0.1M KPi.

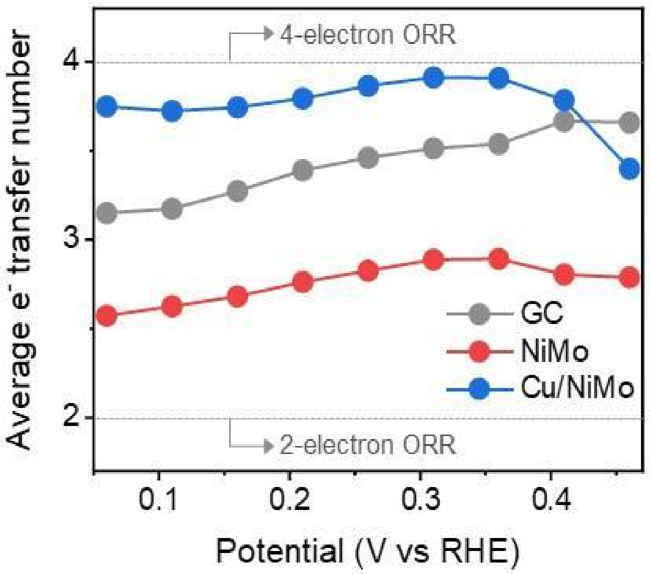

Figure S13. Iodometric titration curve for H_2O_2 with different concentration. The absorbance was measured at 350 nm using the FIA-UV/vis system.

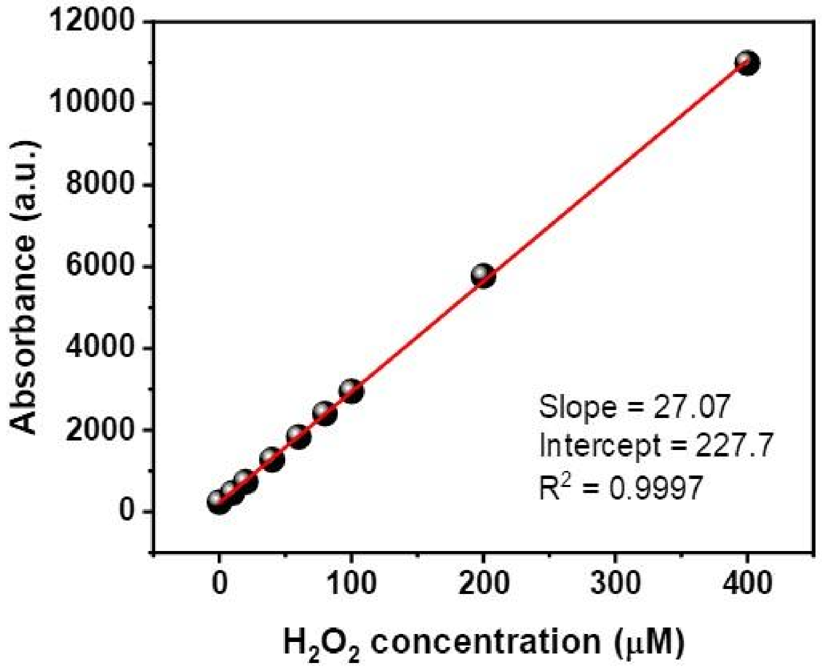

Figure S14. Durability of electrodes with (a) repetitive CV for 1,000 cycles and (b) chrono-voltammetry at -20 mA cm^{-2} for 200+ h.

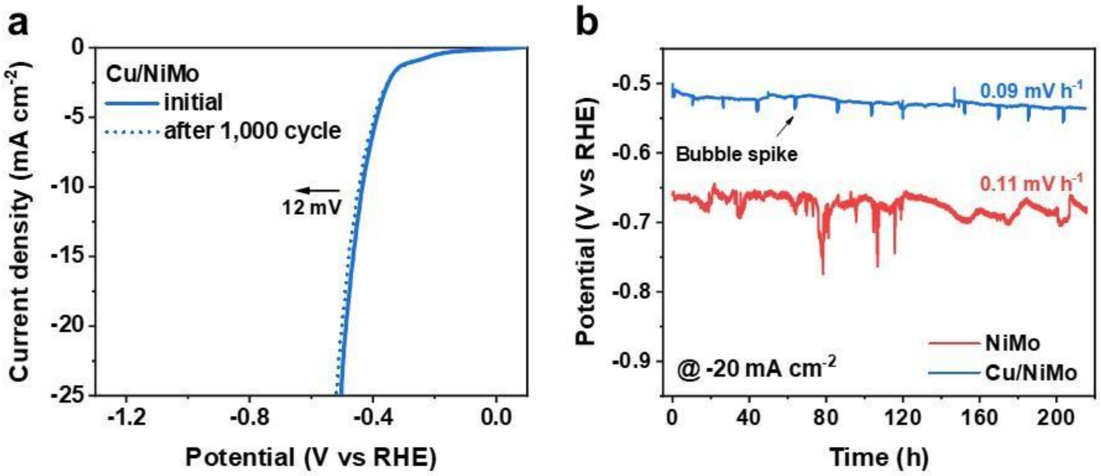

Figure S15. Structural characterization after chrono-amperometry at -20 mA cm^{-2} for 200 hours in 108 mM KPi. FE-SEM and EDS mapping images of (a) NiMo and (b) Cu/NiMo, and (c) thier surface atomic composition.

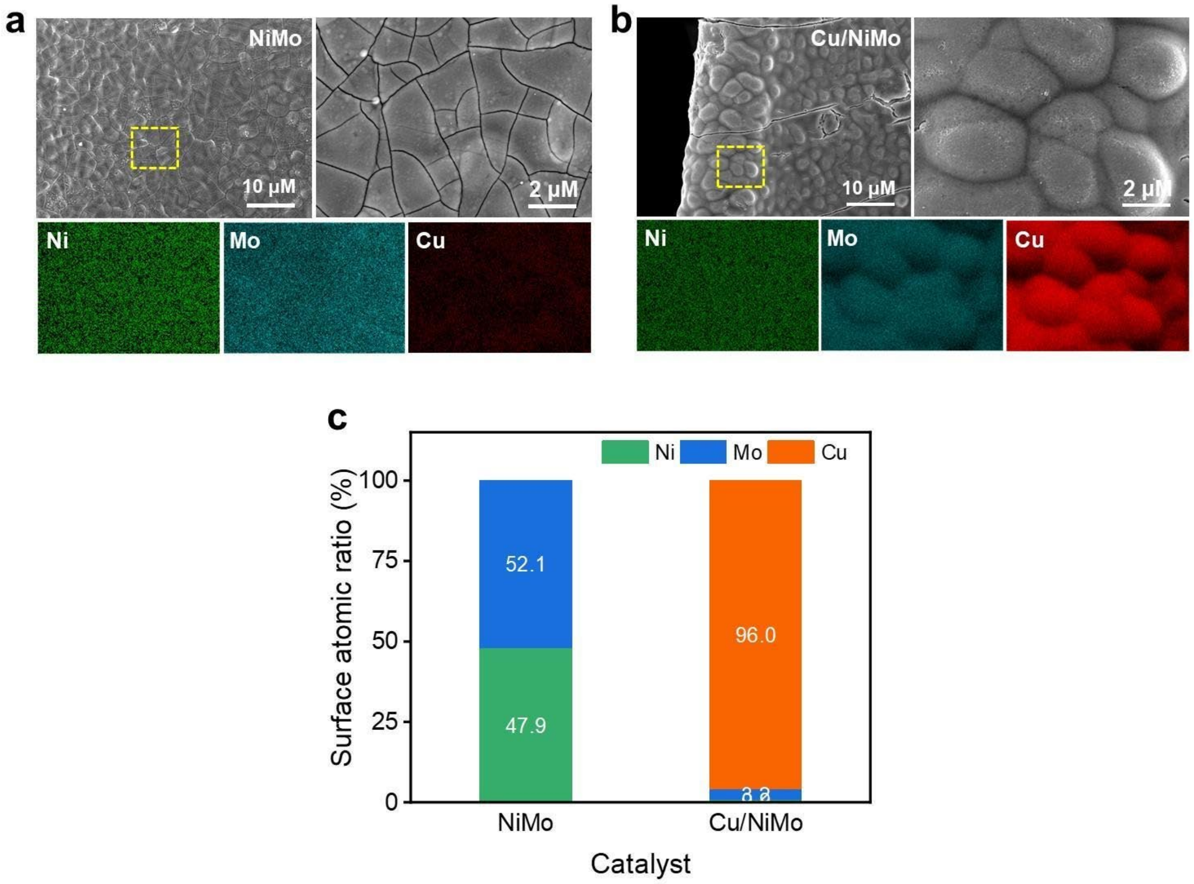

Figure S16. Cross-sectional SEM images and EDS-mapping for Cu/NiMo (a, b) before and (c, d) after chrono-amperometry at -20 mA cm^{-2} for 100 hours in 108 mM KPi.

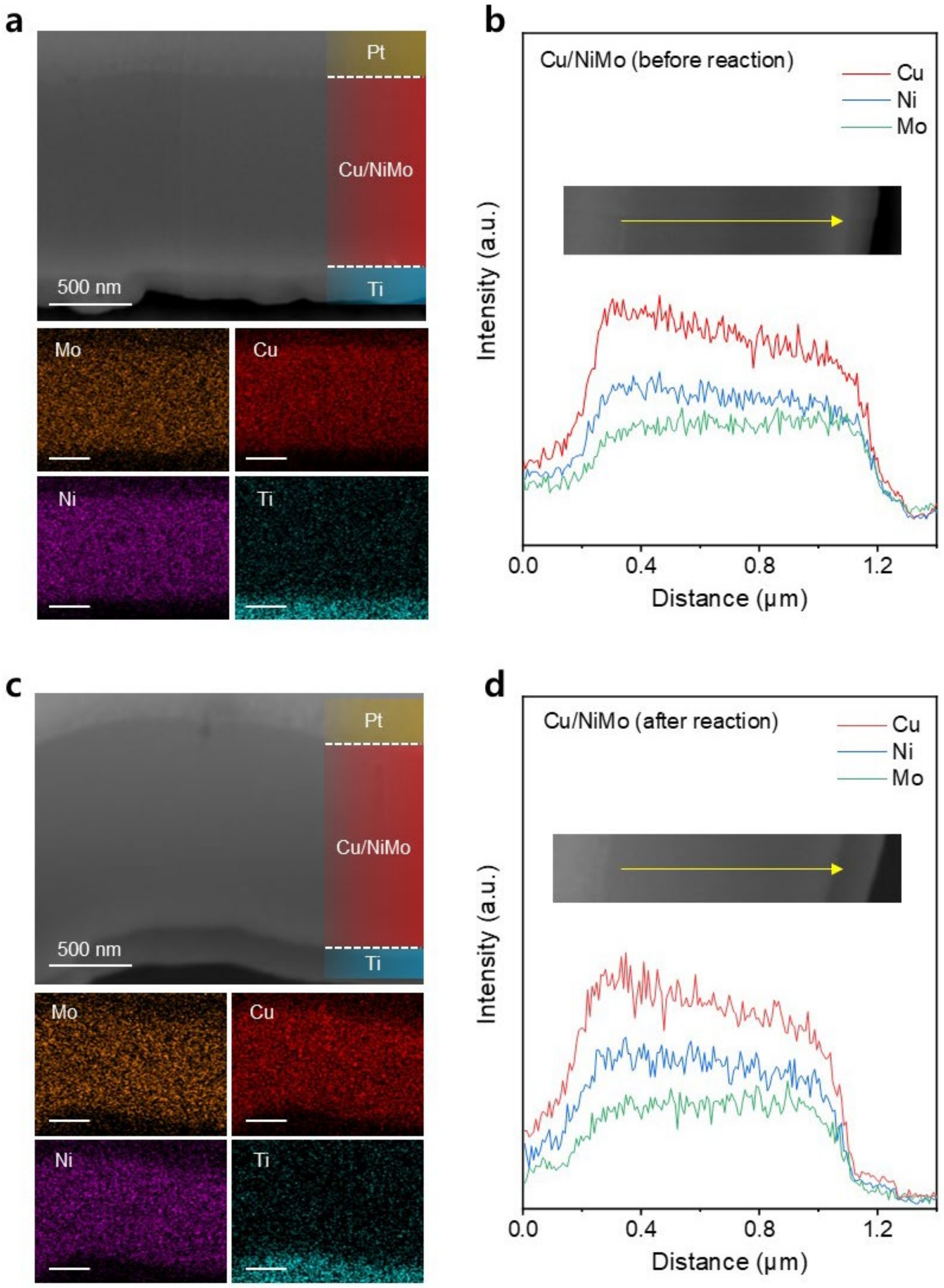

Table S2. Parameters for potentiodynamic polarization measurement on HEC electrodes in 108 mM KPi where E_{corr} is corrosion potential; i_{corr} is corrosion current; βa is anodic Tafel slope; βc is cathodic Tafel slope.

Electrodes	E_{corr} (V vs. Ag/AgCl)	i_{corr} (μA cm^{-2})	βa (mV dec^{-1})	βc (mV dec^{-1})
NiMo	-0.281	1.3	166	124
Cu/NiMo	-0.375	59.8	179	153

Notes: Corrosion potential (E_{corr}) are the potential where the reduction and oxidation reactions occur simultaneously, and the corrosion current is the measured current in the Tafel plots (log i vs. E) where cathodic and anodic Tafel slope meet.

Figure S17. XPS spectra of (a) Ni 2p, (b) Mo 3d, (c) Cu 2p, (d) O 1s, and (e) survey scan after chrono-amperomemtry at -20 mA cm^{-2} for 200 hours in 108 mM KPi.

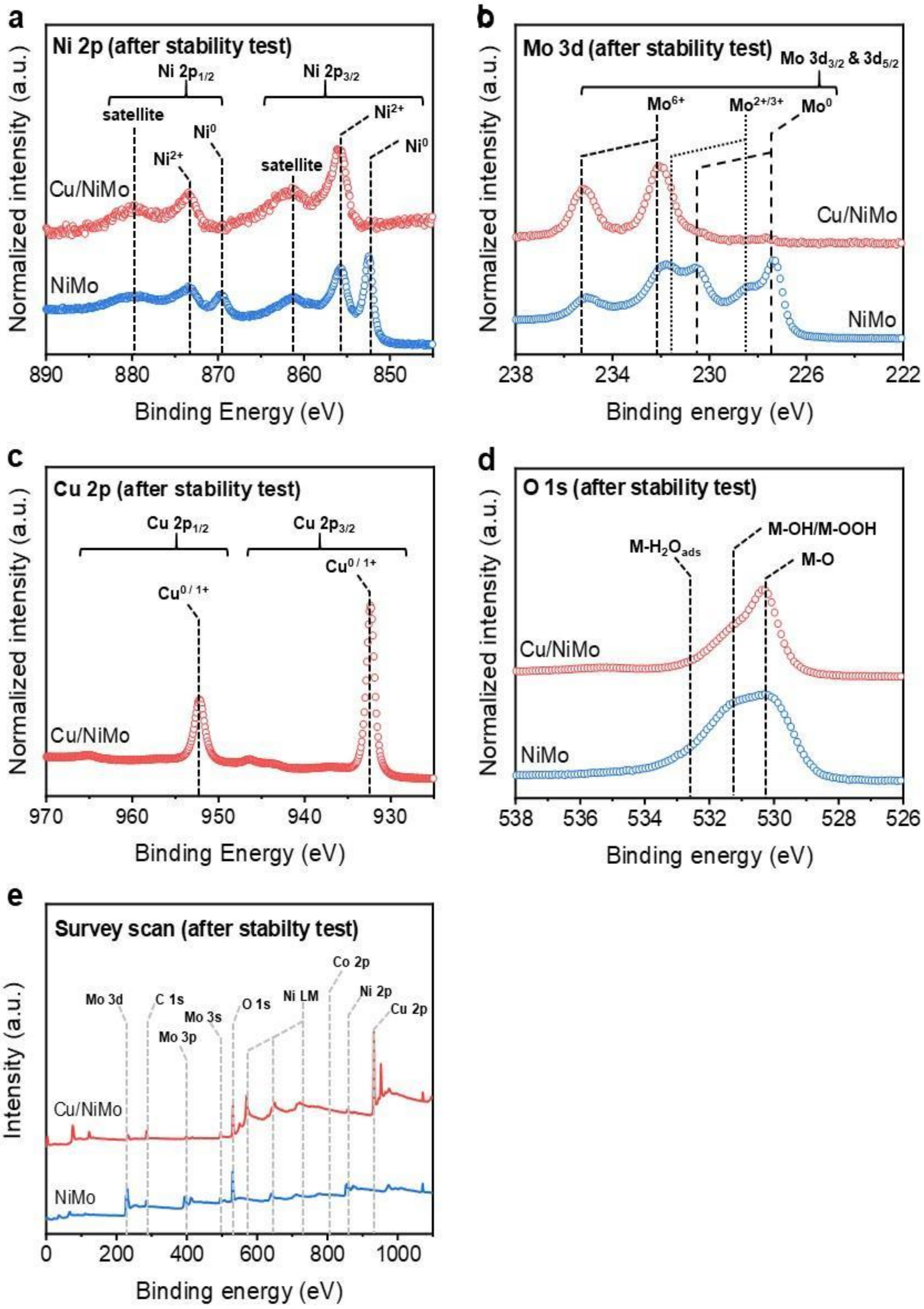

Note : XPS spectra of NiMo and Cu/NiMo electrodes after long-term stability test show that no significant changes in the chemical states of consisting metals were observed.

Figure S18. Microbial fermentation performance in the MES system with the (a–c) Cu/NiMo and (d-f) GC electrode poised at different potentials. (a, d) Microbial cell growth curves with OD_{600}, (b, e) current densities, and (c, f) PHB content (white circle) and titers (bar) of the Cu/NiMo and GC electrodes. Each experiment was performed in triplicate, and the error bars indicate the standard deviation of the mean of triplicate values.

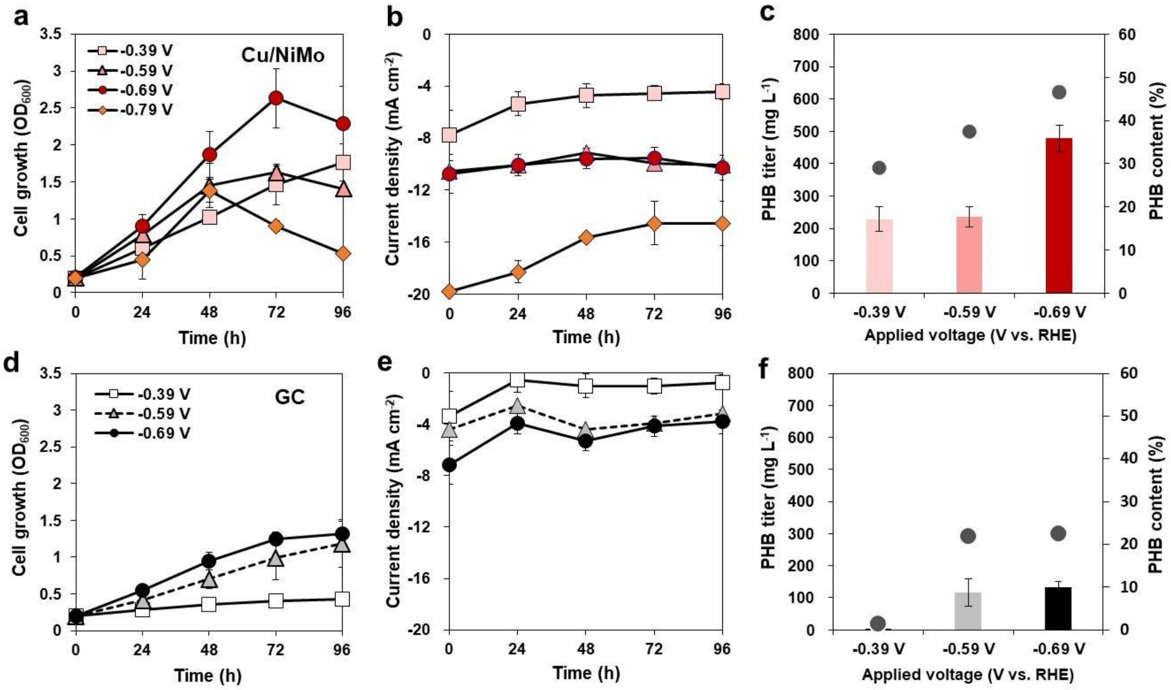

Figure S19. Spot assay of *C. necator* H16 with different concentrations of Ni^{2+}, Co^{2+}, and Cu^{2+}.

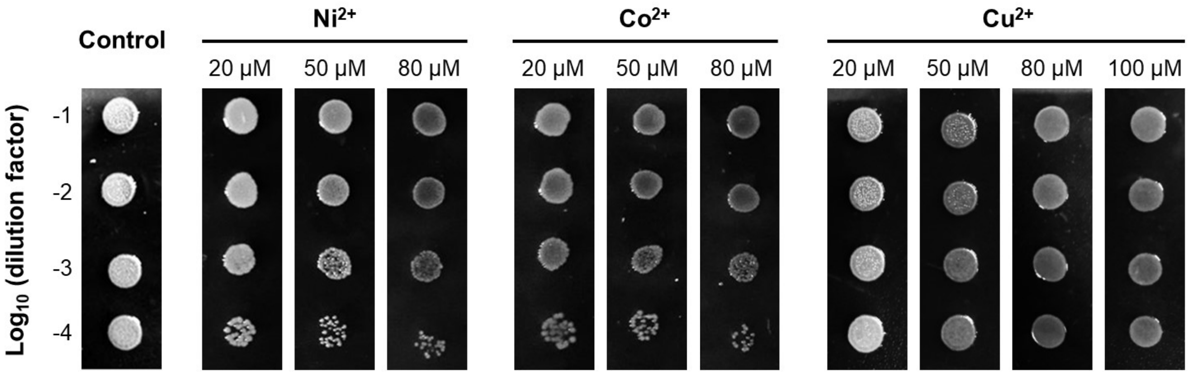

Note: Spot assays were performed to investigate the cytotoxicity of Ni, Co, and Cu cations. Pre-cultures of *C. necator* H16 grown in rich LB medium were washed with minimal medium and suspended to an initial OD_{600} of 0.2. The suspension was diluted 10-fold serially up to 10^{-4} and spotted on the minimal media agar plates containing each toxicant. The spotted plates were then incubated in the pressure vessel containing a gas mixture containing 20% of O_2, 10% of CO_2, and 70% of H_2 for 120 hours. The toxicity on the Ni^{2+} and Co^{2+} were visible at 1/1000 dilution higher than 50 μM and 20 μM, respectively. For Cu^{2+}, insignificant toxicity was observed lower than 100 μM.

Figure S20. Rotating disk voltammetry curves of Pt/C for H_2O_2 decomposition in an Ar-purged 108 mM KPi with 5 mM H_2O_2.

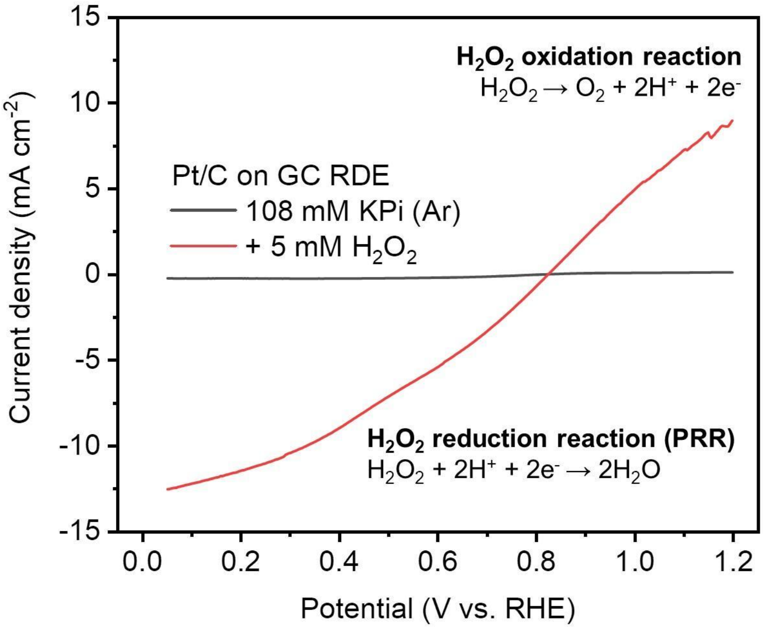

Figure S21. Logarithm plot for the H_2O_2 decomposition curves in 108 mM KPi buffer with 5 mM H_2O_2 at -0.59 V vs. RHE, and the kinetic constants.

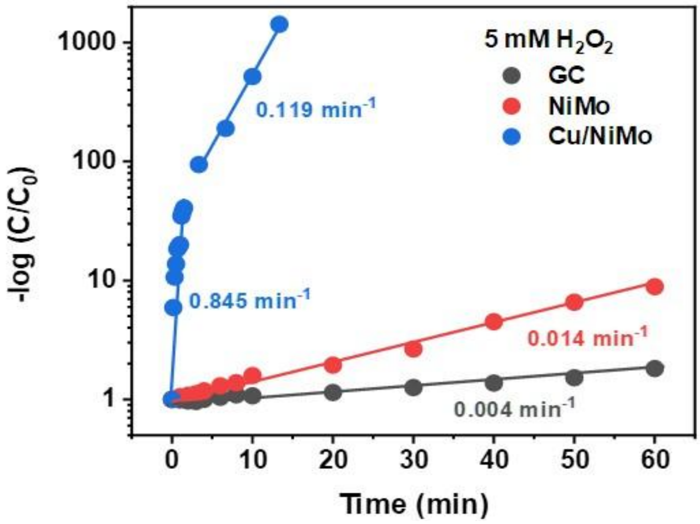

Note: Kinetic constant for H_2O_2 decomposition was determined from the linear slope of the logarithm plot of H_2O_2 decomposition with following equation:

$$ln\,\frac{C_0}{C} = k_{dec}\,t$$

where C_0 is the initial concentration of H_2O_2 (5 mM), C is H_2O_2 concentration determined by iodometric spectrometry, and k_{dec} is the kinetic rate constant for H_2O_2 decomposition. The decomposition rates for Cu/NiMo (0.845 min^{-1}) was considerably higher than those of NiMo (0.014 min^{-1}) and GC (0.004 min^{-1}).

Figure S22. EPR spectra of radical intermediates trapped by DMPO in Ar-sparged 108 mM KPi with 5 mM H_2O_2 with applied potential of -0.59 V vs. RHE for Cu/NiMo electrode. ▼ denotes DMPO–•R adduct.

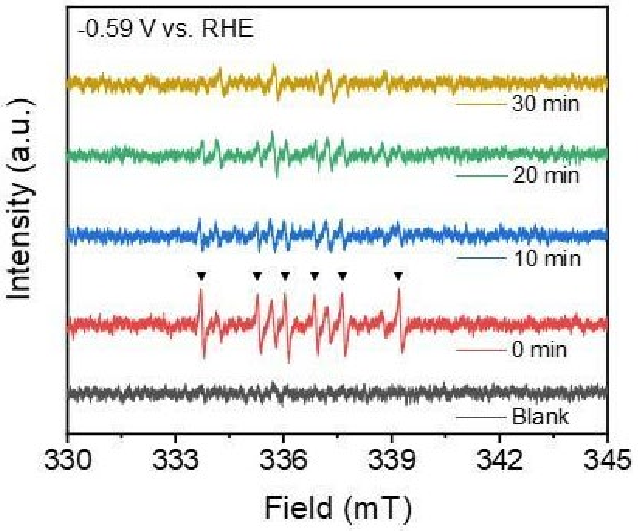

Table S3. Comparison on the reported hydrogen-driven MES system for bioelectrochemical production using *C. necator.*

Organism /strain	Reactor type	Cathode	Applied potential (V vs. RHE)	Current density (mA cm^{-2})	Product	Production		Reference
						Titer (g/L)	Production rate[a] (g/L/d)	
C. necator H16	Single chamber	Cu/NiMo	-0.69	-10	PHB	0.48	0.120	This work
C. necator H16	Single Chamber	NiMo	-0.69	-7.2	PHB	0.30	0.074	This work
C. necator H16	Single Chamber	Glassy carbon	-0.69	-4.9	PHB	0.13	0.034	This work
C. necator H16	Single Chamber	Ni@N-C[b]	~ -0.79	-4.8	PHB	0.38	0.054	[4]
C. necator H16	Single Chamber	Pt/C	~ -0.79	-4.8	PHB	0.41	0.059	[4]
C. necator H16	Single Chamber	NiMoZn	2.7 [c]	~ -2.6	PHB	-	-	[5]
C. necator Re2133-pEG12	Single Chamber	Stainless steel	3.0 [c]	~ -6.7	PHB	0.22	0.04	[5]
C. necator H16	Single Chamber	Co-P alloy	2.0 [c]	-4	PHB	0.70	0.117	[6]
C. necator H16	Double Chamber	Carbon cloth	-0.19	-0.1	PHB	0.16	0.03	[7]

[a] Overall production rate (g/L/d)

[b] A complex of Ni nanoparticles embedded in N-doped carbon nanotubes

[c] Cell voltage in a two electrode configuration vs. cobalt phosphate (CoPi) anode

References

[1] M. Schalenbach, F. D. Speck, M. Ledendecker, O. Kasian, D. Goehl, A. M. Mingers, B. Breitbach, H. Springer, S. Cherevko, K. J. Mayrhofer, *Electrochim. Acta* **2018**, 259, 1154.

[2] A. Jain, S. P. Ong, G. Hautier, W. Chen, W. D. Richards, S. Dacek, S. Cholia, D. Gunter, D. Skinner, G. Ceder, K. a. Persson, *APL Materials* **2013**, 1, 011002.

[3] C. C. L. McCrory, S. Jung, J. C. Peters, T. F. Jaramillo, *Journal of the American Chemical Society* **2013**, 135, 16977.

[4] Z. Li, G. Li, X. Chen, Z. Xia, J. Yao, B. Yang, L. Lei, Y. Hou, *ChemSusChem* **2018**, 11, 2382.

[5] J. P. Torella, C. J. Gagliardi, J. S. Chen, D. K. Bediako, B. Colón, J. C. Way, P. A. Silver, D. G. Nocera, *Proceedings of the National Academy of Sciences of the United States of America* **2015**, 112, 2337.

[6] C. Liu, B. C. Colón, M. Ziesack, P. A. Silver, D. G. Nocera, *Science* **2016**, 352, 1210.

[7] X. Chen, Y. Cao, F. Li, Y. Tian, H. Song, *ACS Catalysis* **2018**, 8, 4429.
